# Intrathecal Contrast-Enhanced Magnetic Resonance Imaging of Cerebrospinal Fluid Dynamics and Glymphatic Enhancement in Idiopathic Normal Pressure Hydrocephalus

**DOI:** 10.3389/fneur.2022.857328

**Published:** 2022-04-06

**Authors:** Per Kristian Eide, Aslan Lashkarivand, Åsmund Aleksander Hagen-Kersten, Øivind Gjertsen, Bård Nedregaard, Ruth Sletteberg, Grethe Løvland, Svein Are Sirirud Vatnehol, Are Hugo Pripp, Lars Magnus Valnes, Geir Ringstad

**Affiliations:** ^1^Department of Neurosurgery, Oslo University Hospital-Rikshospitalet, Oslo, Norway; ^2^Institute of Clinical Medicine, Faculty of Medicine, University of Oslo, Oslo, Norway; ^3^Department of Radiology, Oslo University Hospital-Rikshospitalet, Oslo, Norway; ^4^The Intervention Centre, Oslo University Hospital-Rikshospitalet, Oslo, Norway; ^5^Institute of Optometry Radiography and Lighting Design, Faculty of Health and Social Sciences, University of South Eastern Norway, Drammen, Norway; ^6^Oslo Centre of Biostatistics and Epidemiology, Research Support Services, Oslo University Hospital, Oslo, Norway; ^7^Faculty of Health Sciences, Oslo Metropolitan University, Oslo, Norway; ^8^Department of Geriatrics and Internal Medicine, Sorlandet Hospital, Arendal, Norway

**Keywords:** idiopathic normal pressure hydrocephalus, cerebrospinal fluid, glymphatic function, magnetic resonance imaging, intrathecal gadobutrol, imaging biomarkers

## Abstract

Idiopathic normal pressure hydrocephalus (iNPH) is a neurodegenerative disease, characterized by cerebrospinal fluid (CSF) flow disturbance. Today, the only available treatment is CSF diversion surgery (shunt surgery). While traditional imaging biomarkers typically assess CSF space anatomy, recently introduced imaging biomarkers of CSF dynamics and glymphatic enhancement, provide imaging of CSF dynamics and thereby more specifically reveal elements of the underlying pathophysiology. The biomarkers address CSF ventricular reflux grade as well as glymphatic enhancement and derive from intrathecal contrast-enhanced MRI. However, the contrast agent serving as CSF tracer is administered off-label. In medicine, the introduction of new diagnostic or therapeutic methods must consider the balance between risk and benefit. To this end, we performed a prospective observational study of 95 patients with iNPH, comparing different intrathecal doses of the MRI contrast agent gadobutrol (0.10, 0.25, and 0.50 mmol, respectively), aiming at the lowest reasonable dose needed to retrieve diagnostic information about the novel MRI biomarkers. The present observations disclosed a dose-dependent enrichment of subarachnoid CSF spaces (cisterna magna, vertex, and velum interpositum) with dose-dependent ventricular reflux of tracer in iNPH, as well as dose-dependent glymphatic tracer enrichment. The association between tracer enrichment in CSF and parenchymal compartments were as well dose-related. Intrathecal gadobutrol in a dose of 0.25 mmol, but not 0.10 mmol, was at 1.5T MRI considered sufficient for imaging altered CSF dynamics and glymphatic enhancement in iNPH, even though 3T MRI provided better sensitivity. Tracer enrichment in CSF at the vertex and within the cerebral cortex and subcortical white matter was deemed too low for maintaining diagnostic information from a dose of 0.10 mmol. We conclude that reducing the intrathecal dose of gadobutrol from 0.50 to 0.25 mmol gadobutrol improves the safety margin while maintaining the necessary diagnostic information about disturbed CSF homeostasis and glymphatic failure in iNPH.

## Introduction

Idiopathic normal pressure hydrocephalus (iNPH) is a neurodegenerative disease and a subtype of dementia comprising the symptoms of gait ataxia, urinary incontinence, and cognitive impairment in combination with disturbed cerebrospinal fluid (CSF) homeostasis. Today, the only effective treatment is CSF diversion (shunt) surgery that may improve symptoms, though it remains disputed which should be offered surgery (1). The American-European (2) and Japanese (3) diagnostic criteria are primarily based on the clinical picture and imaging signs of CSF space abnormality where imaging biomarkers address the morphology of the cerebral ventricles. Additionally, the lumbar CSF pressure should be normal to differentiate from other types of hydrocephalus. However, the fulfillment of the clinical and imaging criteria of “probable” or “possible” iNPH does not predict clinical response to shunt surgery (2, 4). To predict whether a symptomatic patient suffers “shunt-responsive iNPH”, supplemental tests have included the assessment of clinical response to CSF drainage of short (Tap test) or long (extended lumbar drain) duration, measurements of the CSF pressure change following fluid infusion to the lumbar or ventricular CSF space (infusion tests) or long-term monitoring of static/pulsatile intracranial pressure (ICP) (4–8). Proper patient selection is worthwhile since shunt surgery may be accompanied by lasting symptom improvement in a substantial proportion of patients (9–11).

There is an increasing awareness that iNPH may be a rather common dementia subtype, possibly affecting more than 5% of individuals above 80 years (12, 13). It is a severe brain disease with high 5-year mortality (14, 15). With an aging population, there is a need for biomarkers that more precisely address the underlying pathophysiology. The established anatomic biomarkers Evan's index, callosal angle, and disproportional enlarged subarachnoid space hydrocephalus (DESH) provide morphological information about CSF space anatomy. However, their ability to predict clinical response to CSF diversion surgery remains disputed (16).

Adding to the established imaging biomarkers, we recently proposed functional imaging biomarkers for iNPH disease, based on imaging of CSF redistribution (degree of ventricular reflux), and imaging of CSF and glymphatic enhancement (17, 18). The association between neurodegeneration and impaired clearance of toxic metabolic by-products from CSF and the brain has recently emerged as a possible crucial mechanism behind iNPH disease (18). Furthermore, there is a clear histological overlap between iNPH and Alzheimer's diseases; both are characterized by deposition within the brain of toxic metabolites such as amyloid-β and tau (19, 20). Patients with positive CSF biomarkers of Alzheimer's, e.g., CSF levels of amyloid-β and tau, responded less to CSF diversion (21). Accordingly, impaired CSF clearance may be of particular significance since there are no known blood-brain-barrier (BBB) transporters for tau, and toxic isoforms of amyloid-β (e.g., pyroglutamate Aβ, pE3-Aβ) are primarily removed along extra-vascular pathways (22). The subarachnoid CSF space communicates directly with the brain interstitial space *via* the perivascular spaces (23, 24). Recently it was proposed that impaired glymphatic clearance of toxic metabolites is a common mechanism for dementia diseases, such as Alzheimer's disease (amyloid-β, tau), and Parkinson's disease (α-synuclein) (25).

The functional imaging biomarkers of CSF dynamics and glymphatic enhancement previously reported by our group (24, 26, 27) require an intrathecal injection of an MRI contrast agent. This may be considered a drawback since MRI contrast agents are used off-label and may be accompanied by neurotoxic effects (28). When new methods are introduced in medicine, there is always a need for determining the balance between risk and benefit to improve the therapeutic index, which also concerns intrathecal MRI contrast agents (29). For intrathecal contrast-enhanced MRI, macrocyclic chelates, e.g., gadobutrol, are preferable as they are more stable than the previous linear contrast agents. Toxic doses have not been reported for intrathecal gadobutrol in doses 1.0 mmol or below (28). We have used intrathecal gadobutrol in a dose of 0.5 mmol with good experience from a safety perspective (30, 31), but we have previously not determined the lowest dose needed to maintain the diagnostic information.

On this background, the present study was undertaken to examine the lowest sufficient dose of intrathecal gadobutrol needed to maintain adequate image quality for clinical assessment of MRI biomarkers of CSF dynamics and glymphatic enhancement in patients with iNPH. Secondarily, we questioned the role of these biomarkers in iNPH pathophysiology.

## Materials and Methods

### Approvals and Study Design

The Regional Committee for Medical and Health Research Ethics (REK) of Health Region South-East, Norway (2015/96), The Institutional Review Board of Oslo university hospital (2015/1868), and The National Medicines Agency (15/04932-7) approved the study. Participants were included after written and oral informed consent. The study was conducted according to ethical standards of the Helsinki Declaration of 1975 (and as revised in 1983).

The study design was prospective and observational, primarily comparing MRI biomarkers of CSF dynamics and glymphatic enhancement in patients with iNPH using different doses of intrathecal gadobutrol (Gadovist, Bayer Pharma AG, Berlin, Germany) as CSF tracer, and secondarily comparing how different MRI biomarkers associate.

### Patients

The study included consecutive patients with iNPH undergoing intrathecal contrast-enhanced MRI and phase-contrast MRI, as part of their neurosurgical work-up within the Department of Neurosurgery at the Oslo University Hospital-Rikshospitalet, Norway, during the six-year period of October 2015 to October 2021. The patients fulfilled the criteria of “probable” iNPH (or “possible” iNPH if no ICP monitoring was performed in our department), according to the American-European guidelines (2). The severity of symptoms was graded according to previously described iNPH scoring of symptom severity, with scores spanning from worst (=3) to best (=15) scores, assessing the combined severity of gait disturbance, urinary incontinence, and dementia (5, 11). It was beyond the scope of this study to examine how MRI biomarkers predict the outcome of shunt surgery.

### MRI

The MRI protocol was standardized, as previously described (24, 27). Sagittal 3D T1-weighted gradient-echo volume scans were obtained using a 3 Tesla (3T) Philips Ingenia MRI scanner (Philips Medical systems, Best, The Netherlands), or a 1.5T Aera Siemens MRI scanner (Siemens Erlangen, Germany). Imaging sequence parameters at 3T were: Repetition time (TR) = ‘shortest' (typically 5.1ms), echo time (TE) = ‘shortest' (typically 2.3 ms), flip angle (FA) = 8, and voxel size 1 mm^3^. T1 imaging sequence parameters (T1 MPRAGE) at 1.5T were: TR = 1,900 ms, TE = 2.36 ms and inversion time (TI) = 900 ms, FA = 10 and with voxel size 1 mm^3^. Equal MRI protocol settings were used at each scanner before (Baseline), and 24 and 48 h after the intrathecal injection of gadobutrol. At 3T, T1 imaging was also carried out after intrathecal contrast administration on Day 1. We first included patients who were examined in a 3T MRI scanner; they received intrathecal gadobutrol in a dose of 0.5 mmol only. Secondly, we included patients who were examined in a 1.5T MRI scanner; they received intrathecal gadobutrol in the alternating doses of 0.10, 0.25, or 0.5 mmol. For logistic reasons, 1.5T imaging at Day 1 after intrathecal gadobutrol was not feasible.

At both 3T and 1.5T, we also obtained 3D T2 fluid-attenuated inversion recovery (FLAIR) scans. The image parameters at 3T were: TR = 4,800 ms, TE = ‘shortest' (typically 318 ms), TI = 1,650 ms, with voxel size 1 mm^3^. Image parameters at 1.5T were: TR = 5,000 ms, TR = 337 ms, TI = 1,600, FA = 120 and with voxel size 1 mm^3^. In the present study, FLAIR scans were used for assessing Fazeka's grade.

### MRI Biomarkers of CSF Dynamics and Glymphatic Enhancement

The MRI biomarkers of CSF flow include two measures:

(a) Estimation of tracer clearance from CSF spaces 24 and 48 h after intrathecal contrast (gadobutrol) administration. For each time point, circular regions of interest (ROIs) were placed on 1 mm thick slices within the CSF of cisterna magna and within a cerebral sulcus underneath the vertex where partial averaging with brain tissue could be avoided, preferably the central sulcus. At the individual level, ROI positions were identical between time points. Measurements were done directly in the hospital Picture archiving and communication system (PACS) (Sectra IDS7, Sectra, Sweden), where each ROI provides the mean T1 signal intensity (in signal units) from the image greyscale. For comparison, we also included the CSF space of the velum interpositum, estimated from FreeSurfer software, which represents an approximately mid-level position between the vertex region and cisterna magna.

(b) We have previously introduced a grading of ventricular reflux of CSF tracer as a marker of pathological CSF redistribution (17, 18). From T1 weighted images, ventricular reflux was graded at 24 h after intrathecal MRI contrast agent administration as follows: Grade 0: No supra-aqueductal reflux. Grade 1: Any sign of transient supra-aqueductal reflux at Day 1. Grade 2: Transient enrichment of lateral ventricles at Day 1. Grade 3: Lasting enrichment of lateral ventricles Day 2 (but not isointense with subarachnoid CSF). Grade 4: Lasting enrichment of lateral ventricles at Day 2 (isointense with subarachnoid CSF). In the current imaging protocol, imaging was not obtainable post-contrast on Day 1 at 1.5T for logistic reasons. This was acceptable as we only consider grades 3–4 on Day 2 indicative of abnormal reflux in iNPH. Therefore, the assessment of grades 1–2 was not examined in this study.

The MRI biomarkers of glymphatic enhancement rely on estimating enrichment of the CSF tracer within extra-vascular brain parenchyma at defined time points after intrathecal CSF tracer administration, as previously described (27). In short, we applied FreeSurfer software (version 6.0) (http://surfer.nmr.mgh.harvard.edu/) for the segmentation, parcellation, and registration/alignment of the longitudinal data, and to determine the tracer-induced increase in T1 signal intensity (32). Using a hybrid watershed/surface deformation procedure (33), non-brain tissue is removed, followed by the segmentation of the subcortical white matter and deep gray matter structures (including the hippocampus, amygdala, caudate, putamen, and ventricles) (34, 35). The MR images of each patient were used to create a median template registered to the baseline (36), and for each patient, the MR images were registered to the corresponding template applying a rigid transformation (36). The registrations were checked manually to correct any registration errors. Adjustments for changes in the gray-scale between MRI scans were made by dividing the T1 signal unit for each time point by the T1 signal unit of a reference region of interest (ROI) for the respective time point placed within the posterior part of the orbit (37). This *normalized T1 signal unit* corrects for baseline changes of image greyscale due to automatic image scaling. For visualization, a median template image of each patient group was created for each time point, and a relative change in intensity from before intrathecal gadobutrol to 24 h after gadobutrol was computed. The image was constructed by using the median value of each segmented region, and subsequent using the median of the cohort.

### Criteria for Assessing the Lowest Acceptable Dose of Intrathecal Gadobutrol

The criteria for the lowest possible dose of intrathecal gadobutrol refer to the lowest dose needed to maintain necessary diagnostic information: (1) Tracer enrichment in CSF at vertex was obligatory since tracer enrichment in CSF is a requirement for glymphatic enhancement. We previously reported a significant correlation between enrichment in CSF and nearby brain parenchyma (24, 26), and between CSF and nearby parasagittal dura (38). (2) Ventricular tracer enrichment allowing for reliable assessment of ventricular reflux grade. (3) Tracer enrichment in parenchyma allowing for reliable assessment of glymphatic enhancement in brain parenchyma.

### MRI Biomarkers of CSF Space Morphology and Neuro-Degeneration

Following our standardized protocol, three MRI biomarkers of CSF space morphology were determined: (a) Evans' index was determined from T1-weighted axially reconstructed images in a plane parallel to a plane defined by a line between the anterior and posterior commissures (AC-PC plane), respectively (1 mm thickness), which is the dividend between the largest diameter of the frontal horns and the largest inner diameter of the cranium in the same slice (39). (b) The callosal angle was measured on T1-weighted coronal images perpendicular to the AC-PC plane, representing the angle between lateral ventricles at the level of the posterior commissure (40). (c) The DESH (disproportional enlarged subarachnoid space hydrocephalus) sign (41) was assessed on T1-weighted coronal images and scored as yes/no; the DESH sign is the combination of 1. Enlarged ventricles; 2. Widening of Sylvian fissure; 3. Tight sulci at upper/medial cerebral convexities.

Our routine further included the determination of three MRI biomarkers of neurodegeneration: (a) The Schelten's score (42) for medial temporal atrophy (MTA) is a visual rating of the width of the choroid fissure, the width of the temporal horn, and the height of the hippocampal formation [Score 0 (no atrophy), score 1 (only widening of choroid fissure), score 2 (also widening of the temporal horn of lateral ventricle), score 3 (moderate loss of hippocampal volume, decrease in height), and score 4 (severe volume loss of hippocampus)]. (b) The Fazeka's scale for white matter lesions (43) includes four scores and was assessed at FLAIR [Score 0: None or a single punctate white matter hyperintensity lesion. Score 1: Multiple punctate lesions. Score 2: The beginning confluence of lesions (bridging). Score 3: Large confluent lesions]. (c) Entorhinal cortex thickness was determined on coronally reconstructed T1 volume acquisitions with 1 mm slice thickness at the level of the hippocampal sulcus and measured from the entorhinal cortex surface to the gray/white matter interface, and midway between the tentative location of parasubiculum and perirhinal cortex, as previously described (26).

### Statistical Analyses

Statistical analyses were performed using SPSS version 27 (IBM Corporation, Armonk, NY, USA) and Stata/SE 16.1 (StataCrop LLC, College Station, TX, USA).

Continuous data were presented as mean (SD) or mean (95% CI), as appropriate. Group difference between categorical or continuous data was assessed with Pearson Chi-square test or independent samples *t*-test, respectively. Repeated measurements were examined with linear mixed models by maximum likelihood estimation using a subject-specific random intercept. Using the estimated marginal mean from the statistical model, we tested the difference between the individuals with different intrathecal doses and 1.5T or 3T MRI at each time point. The normal distribution assumptions were assessed with descriptive statistics, boxplots, and histograms. It was also conducted for other data in both groups.

Statistical significance was accepted at the 0.05 level (two-tailed).

## Results

### Patient Material

The study was performed from October 2015 to October 2021 and included 95 patients with iNPH who fulfilled the diagnostic criteria of “Probable” iNPH (or “Possible” iNPH if ICP was not measured in our department), according to the American-European guidelines (2). Demographic and clinical information about the patients is shown in Table 1. The patients who received different doses of intrathecal gadobutrol (1.5T MRI: 0.1 mmol, *n* = 18;0.25 mmol, *n* = 25; 0.50 mmol, *n* = 19. 3T MRI: 0.50 mmol, *n* = 33) were comparable, except for significant differences in iNPH scores between some groups (Table 1).

**Table 1:** Demographic and clinical information about the different treatment groups.

	**Total material**	**Intrathecal gadobutrol dosage groups**				
		**1.5T MRI**			**3T MRI**	**Significance**
		**0.10 mmol**	**0.25 mmol**	**0.50 mmol**	**0.50 mmol**	
*N*	95	18	25	19	33	
Sex (F/M; *N*)	36/59	6/12	14/11	7/12	9/24	ns
Age (years)	71.7 ± 5.8	72.3 ± 5.3	72.3 ± 6.2	71.7 ± 4.4	70.8 ± 6.5	ns
Body mass index (kg/m^2^)	27.3 ± 4.6	27.5 ± 5.3	27.6 ± 5.3	27.4 ± 4.3	27.0 ± 4.2	ns
**Clinical grade**
Symptom duration (years)	3.2 ± 2.6	3.4 ± 3.1	2.8 ± 2.0	3.2 ± 2.4	3.3 ± 2.8	ns
Pre-shunt NPH-score^a^	11 (6–14)	11 (9–12)	11 (6–14)	11 (9–13)	12 (8–14)	^b^P <0.05
Gait sub-score	3 (2–4)	4 (3–4)	3 (2–4)	3 (2–4)	4 (3–4)	ns
Incontinence sub-score	4 (1–5)	4 (3–4)	4 (1–5)	4 (3–5)	4 (2–5)	^c^P <0.05
Dementia sub-score	4 (2–5)	4 (3–4)	4 (2–5)	4 (3–5)	4 (3–5)	ns
**Tests of cognitive function**
Mini-mental state (MMS)	27 (14–30)	26 (16–30)	28 (17–30)	27 (20–30)	27 (14–30)	ns

*Categorical data presented as numbers; continuous data presented as mean ± standard deviation, NPH-scores and MMS presented as median (ranges in parentheses). Significant differences between dosage groups were determined by the Pearson Chi-square test for categorical data and by ANOVA with Bonferroni post-hoc tests for continuous data*.

a *NPH-score refers to our previously published grading of NPH symptoms (5)*.

b *0 mmol/1.5T MRI and 0.25 mmol/1.5T MRI vs. 0.50 mmol/3T MRI*.

c *0.10 mmol and 0.25 mmol vs. 3T MRI.50 mmol. Ns, Non-significant*.

The MRI biomarkers of ventriculomegaly and neurodegeneration were comparable across the dosage groups (Table 2). Notably, the estimation of entorhinal cortex thickness differed between the groups examined in the 1.5T vs. 3T MRI scanners (Table 2), probably related to the higher signal-to-noise ratio in the 3T vs. the 1.5T MRI and thereby better distinction of the gray-/white matter interface.

**Table 2:** MRI biomarkers of CSF dynamics, ventriculomegaly and neurodegeneration for the different treatment groups.

	**Total material**	**Intrathecal gadobutrol dosage groups**				
			**1.5T MRI**			**3T MRI**	**Significance**
			**0.10 mmol**	**0.25 mmol**	**0.50 mmol**	**0.50 mmol**	
**CSF dynamics**							
Ventricular reflux	Grade 0	3 (3%)	2 (13%)	–	1 (6%)	–	^a^*P =* 0.01
	Grade 1	2 (2%)	–	–	–	2 (6%)	
	Grade 2	1 (1%)	–	–	–	1 (3%)	
	Grade 3	46 (52%)	12 (80%)	13 (54%)	11 (65%)	10 (30%)	
	Grade 4	37 (42%)	1 (7%)	11 (46%)	5 (29%)	20 (61%)	
**CSF space anatomy**							
Evans index		0.38 ± 0.04	0.37 ± 0.03	0.38 ± 0.05	0.38 ± 0.03	0.38 ± 0.04	ns
Callosal angel		68.6 ± 20.1	63.2 ± 15.8	67.7 ± 18.2	68.1 ± 18.1	72.0 ± 24.2	ns
DESH (Present/Absent; %)		57/92 (62%)	10/15 (67%)	16/25 (64%)	14/19 (74%)	17/33 (52%)	ns
**Neurodegeneration biomarkers**
Scheltens MTA	Grade 0	–	–	–	–	–	ns
	Grade 1	5 (5%)	1 (7%)	1 (4%)	1 (5%)	2 (6%)	
	Grade 2	58 (63%)	7 (47%)	14 (56%)	12 (63%)	25 (76%)	
	Grade 3	29 (32%)	7 (47%)	10 (40%)	6 (32%)	6 (18%)	
Fazekas scale	Grade 0	7 (7.6%)	–	1 (4%)	3 (16%)	3 (9%)	ns
	Grade 1	32 (34.8%)	4 (27%)	10 (40%)	8 (42%)	10 (30%)	
	Grade 2	33 (35.9%)	7 (47%)	10 (40%)	5 (26%)	11 (33%)	
	Grade 3	20 (21.7%)	4 (27%)	4 (16%)	3 (16%)	9 (27%)	
Entorhinal Cortex Thickness (mm)		2.1 ± 0.3	2.1 ± 0.3	2.2 ± 0.2	2.3 ± 0.3	2.0 ± 0.4	^b^P <0.001 ^c^*P =* 0.018

*CSF, Cerebrospinal fluid; DESH, Disproportional enlarged subarachnoid space hydrocephalus; ERC, entorhinal cortex; MRI, magnetic resonance imaging; MTA, medial temporal atrophy. Significant differences between groups were determined by Pearson Chi-square test for categorical data and by ANOVA with Bonferroni post hoc tests for continuous data*:

a *0.1 mmol/1.5T MRI vs.0.25 mmol/1.5T MRI*;

b *0.5 mmol/1.5T MRI vs. 0.5 mmol/3TMRI*;

c *25 mmol/1.5T MRI vs. 0.50 mmol/3T MRI*.

In this cohort of 95 patients with iNPH, it should be noted that ventricular reflux grade 3–4 was present in 83/89 (93%) of patients, an MTA score of 3 was seen in 29/92 (32%) of patients, and a Fazekas score of 3 in 20/92 (22%) of patients.

### Tracer Enrichment Within the Subarachnoid CSF Space

**Figure 1:**
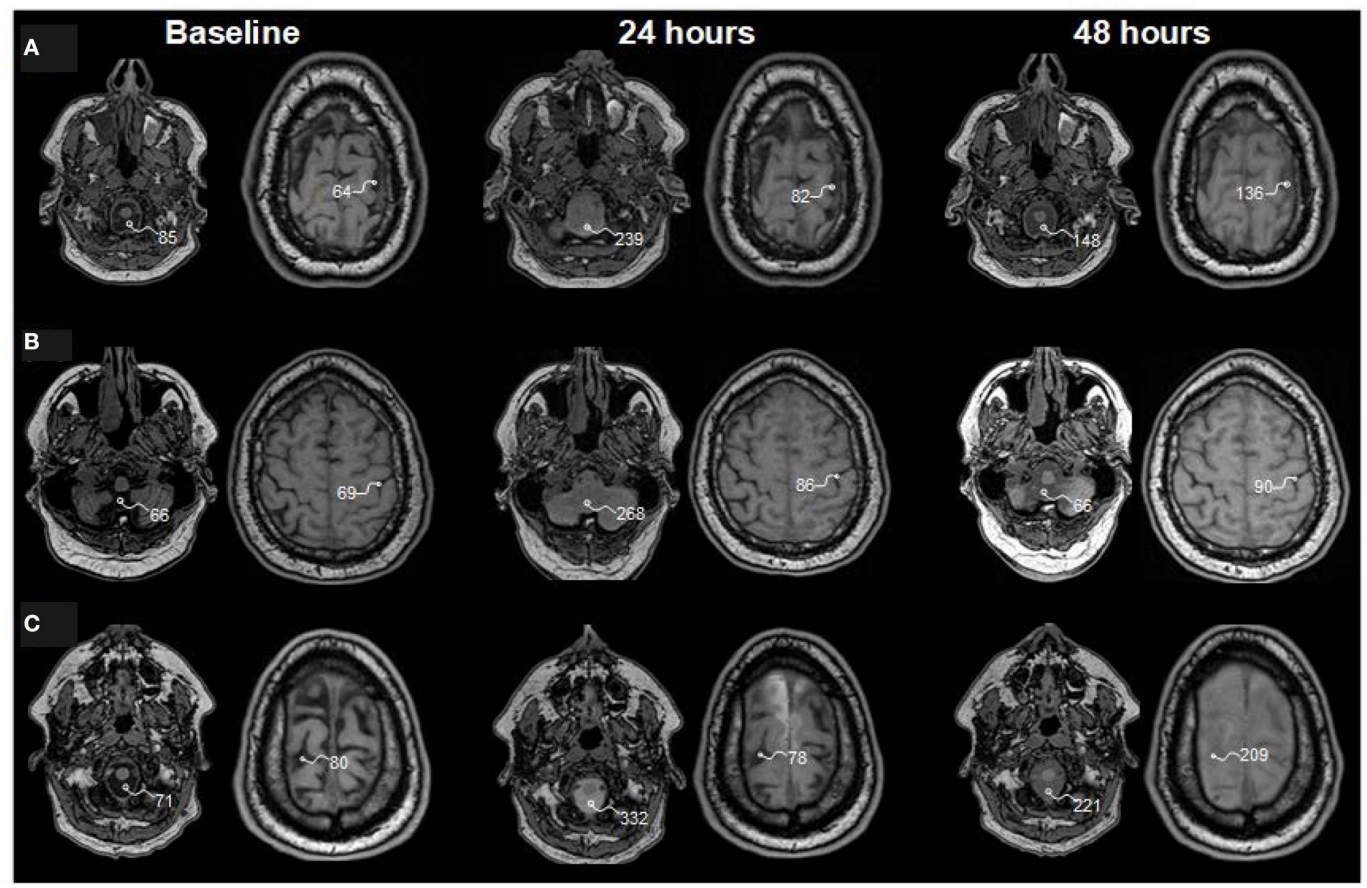
Tracer enrichment within subarachnoid cerebrospinal fluid (CSF) spaces at baseline and 24 and 48 h after intrathecal injection of gadobutrol in doses of **(A)** 0.10 mmol, **(B)** 0.25 mmol, and **(C)** 0.50 mmol. Reconstructed axial T1 weighted images from three patients with idiopathic normal pressure hydrocephalus (iNPH) who were examined in a 1.5T MRI scanner. The T1 signal unit values within regions of interest in cisterna magna and at the vertex are shown and exemplify the strongest increase of T1 signal units after intrathecal gadobutrol in the dose of 0.5 mmol. For analysis of change in T1 signal units, we further normalize the T1 signal units against a region of interest placed within the posterior part of the orbit (not shown here), which provides normalized signal units (37). See Tables 3, 4.

**Figure 2:**
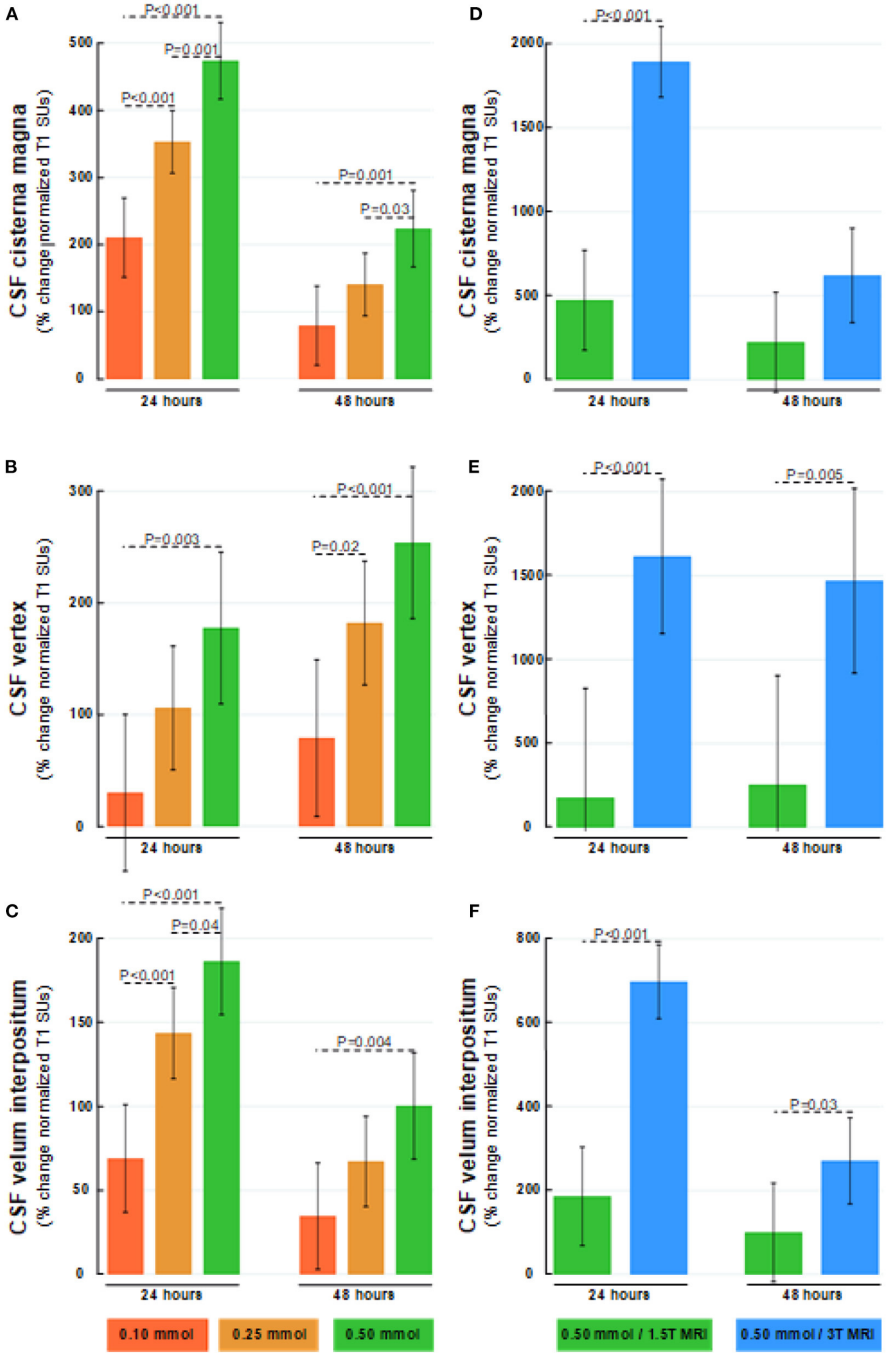
Dose-dependent percentage changes in normalized T1 signal (1.5T MRI scanner) after 24 and 48 h are shown for **(A)** CSF of cisterna magna, **(B)** CSF at the vertex, and **(C)** CSF within velum interpositum calculated from FreeSurfer software following intrathecal gadobutrol in doses of 0.10 mmol (red bars), 0.25 mmol (orange bars), and 0.50 mmol (green bars). The normalized T1 signal at 24 and 48 h after intrathecal gadobutrol (0.50 mmol) within **(D)** CSF of cisterna magna, **(E)** CSF at the vertex, and **(F)** CSF within velum interpositum are shown for 1.5T (green bars) and 3T MRI scanners (blue bars). The bars show mean and 95% confidence intervals. Differences between groups were determined by mixed model analysis.

Intrathecal gadobutrol enriched the CSF of the subarachnoid space, which is visualized as the increase in the T1 signal units. Figure 1 shows in three patients with iNPH the changes in T1 signal units before normalization within CSF regions of interest at cisterna magna and vertex after different doses of intrathecal gadobutrol. The dose-dependent enrichment of CSF spaces is further presented in Figure 2 as the percentage change in normalized T1 signal at 24 and 48 h within CSF of cisterna magna (Figure 2A), vertex (Figure 2B), and velum interpositum (Figure 2C). Table 3 further presents for the different regions the signal units, as well as signal unit ratio and percentage change after 24 and 48 h from before contrast. The tracer enrichment in CSF at vertex after intrathecal gadobutrol in a dose of 0.10 mmol was deemed too low (Figure 2C). There were significant differences for the different doses of intrathecal gadobutrol (0.10, 0.25, or 0.50 mmol), and marked differences in tracer enrichment within CSF between 1.5T and 3T MRI scanners for the same dose of intrathecal gadobutrol (0.50 mmol) (Figures 2D–F).

**Table 3:** Dose-dependent change in T1 signal units and normalized T1 signal within some regions of interest.

	**0.10 mmol/1.5T MRI**										
	**Pre**			**24 h**				**48 h**			
**Anatomical region**	**ROI**	**REF**	**SU-ratio**	**ROI**	**REF**	**SU-ratio**	**%Change**	**ROI**	**REF**	**SU-ratio**	**%Change**
CSF cisterna magna	85 ± 17	386 ± 59	0.22 ± 0.04	253 ± 54	385 ± 56	0.67 ± 0.15	210 ± 89	156 ± 44	397 ± 61	0.39 ± 0.08	79 ± 41
CSF vertex	80 ± 19	386 ± 59	0.21 ± 0.04	101 ± 43	385 ± 56	0.26 ± 0.09	30 ± 50	144 ± 43	397 ± 61	0.36 ± 0.10	79 ± 47
CSF velum interpositum	128 ± 26	386 ± 59	0.33 ± 0.05	215 ± 50	385 ± 56	0.56 ± 0.12	70 ± 35	174 ± 32	397 ± 61	0.44 ± 0.08	35 ± 20
4th ventricle	100 ± 13	386 ± 59	0.26 ± 0.04	222 ± 46	385 ± 56	0.58 ± 0.11	125 ± 36	160 ± 34	397 ± 61	0.40 ± 0.07	57 ± 30
3rd ventricle	93 ± 11	386 ± 59	0.24 ± 0.02	206 ± 54	385 ± 56	0.54 ± 0.13	123 ± 123	150 ± 36	397 ± 61	0.38 ± 0.08	57 ± 33
Lateral ventricles	93 ± 11	386 ± 59	0.24 ± 0.02	182 ± 53	385 ± 56	0.47 ± 0.12	98 ± 50	140 ± 33	397 ± 61	0.35 ± 0.07	48 ± 32
Cerebral cortex	221 ± 27	386 ± 59	0.58 ± 0.05	240 ± 31	385 ± 56	0.63 ± 0.05	9 ± 7	241 ± 32	397 ± 61	0.61 ± 0.05	6 ± 4
Cerebral white matter	282 ± 35	386 ± 59	0.73 ± 0.05	289 ± 34	385 ± 56	0.76 ± 0.05	4 ± 4	297 ± 39	397 ± 61	0.75 ± 0.06	3 ± 3
	**0.25 mmol/1.5T MRI**										
CSF cisterna magna	80 ± 12	365 ± 44	0.22 ± 0.03	368 ± 77	382 ± 49	0.98 ± 0.22	353 ± 115	200 ± 44	389 ± 56	0.52 ± 0.12	141 ± 56
CSF vertex	69 ± 16	365 ± 44	0.19 ± 0.04	136 ± 80	382 ± 49	0.37 ± 0.24	106 ± 158	195 ± 63	389 ± 56	0.52 ± 0.20	182 ± 123
CSF velum interpositum	120 ± 18	365 ± 44	0.33 ± 0.05	304 ± 57	382 ± 49	0.81 ± 0.18	145 ± 53	211 ± 36	389 ± 56	0.55 ± 0.11	67 ± 32
4th ventricle	94 ± 14	365 ± 44	0.26 ± 0.03	334 ± 69	382 ± 49	0.89 ± 0.21	244 ± 76	202 ± 41	389 ± 56	0.53 ± 0.11	105 ± 42
3rd ventricle	87 ± 9	365 ± 44	0.24 ± 0.02	320 ± 73	382 ± 49	0.85 ± 0.22	252 ± 84	192 ± 42	389 ± 56	0.50 ± 0.12	108 ± 45
Lateral ventricles	86 ± 8	365 ± 44	0.24 ± 0.02	274 ± 61	382 ± 49	0.72 ± 0.17	210 ± 66	177 ± 39	389 ± 56	0.46 ± 0.10	96 ± 42
Cerebral cortex	200 ± 30	365 ± 44	0.55 ± 0.07	253 ± 29	382 ± 49	0.67 ± 0.08	23 ± 19	246 ± 28	389 ± 56	0.64 ± 0.07	17 ± 15
Cerebral white matter	260 ± 29	365 ± 44	0.71 ± 0.05	295 ± 28	382 ± 49	0.78 ± 0.06	9 ± 5	297 ± 32	389 ± 56	0.77 ± 0.06	8 ± 5
	**0.50 mmol/1.5T MRI**										
CSF cisterna magna	82 ± 16	382 ± 42	0.22 ± 0.03	475 ± 183	389 ± 42	1.21 ± 0.44	473 ± 222	276 ± 101	403 ± 43	0.69 ± 0.26	224 ± 127
CSF vertex	68 ± 11	382 ± 42	0.18 ± 0.03	188 ± 165	389 ± 42	0.47 ± 0.39	178 ± 223	250 ± 119	403 ± 43	0.62 ± 0.29	254 ± 159
CSF velum interpositum	121 ± 21	382 ± 42	0.32 ± 0.04	345 ± 139	389 ± 42	0.90 ± 0.38	186 ± 143	250 ± 82	403 ± 43	0.63 ± 0.22	100 ± 79
4th ventricle	102 ± 15	382 ± 42	0.27 ± 0.04	375 ± 160	389 ± 42	0.97 ± 0.42	269 ± 174	249 ± 88	403 ± 43	0.63 ± 0.23	136 ± 91
3rd ventricle	91 ± 8	382 ± 42	0.24 ± 0.01	359 ± 162	389 ± 42	0.93 ± 0.44	290 ± 191	235 ± 91	403 ± 43	0.59 ± 0.24	148 ± 105
Lateral ventricles	92 ± 14	382 ± 42	0.24 ± 0.03	313 ± 140	389 ± 42	0.81 ± 0.38	247 ± 175	214 ± 80	403 ± 43	0.54 ± 0.21	128 ± 96
Cerebral cortex	214 ± 28	382 ± 42	0.56 ± 0.05	265 ± 40	389 ± 42	0.68 ± 0.09	23 ± 24	272 ± 33	403 ± 43	0.68 ± 0.07	22 ± 19
Cerebral white matter	276 ± 30	382 ± 42	0.72 ± 0.05	301 ± 28	389 ± 42	0.78 ± 0.06	7 ± 8	319 ± 30	403 ± 43	0.79 ± 0.05	10 ± 8
	**0.50 mmol/3T MRI**										
CSF cisterna magna	14 ± 6	187 ± 89	0.07 ± 0.02	254 ± 125	185 ± 90	1.41 ± 0.48	1893 ± 844	131 ± 73	241 ± 130	0.59 ± 0.32	810 ± 584
CSF vertex	10 ± 5	187 ± 89	0.05 ± 0.02	146 ± 139	185 ± 90	0.79 ± 0.67	1616 ± 1741	165 ± 154	241 ± 130	0.70 ± 0.55	1295 ± 1157
CSF velum interpositum	32 ± 18	187 ± 89	0.17 ± 0.03	235 ± 127	185 ± 90	1.29 ± 0.43	698 ± 338	154 ± 78	241 ± 130	0.69 ± 0.26	328 ± 197
4th ventricle	18 ± 10	187 ± 89	0.10 ± 0.02	224 ± 117	185 ± 90	1.24 ± 0.46	1242 ± 583	114 ± 48	241 ± 130	0.53 ± 0.26	510 ± 333
3rd ventricle	20 ± 11	187 ± 89	0.10 ± 0.01	225 ± 122	185 ± 90	1.25 ± 0.48	1137 ± 528	117 ± 53	241 ± 130	0.55 ± 0.28	460 ± 313
Lateral ventricles	14 ± 8	187 ± 89	0.07 ± 0.02	168 ± 95	185 ± 90	0.94 ± 0.44	1534 ± 818	79 ± 41	241 ± 130	0.39 ± 0.25	556 ± 474
Cerebral cortex	59 ± 32	187 ± 89	0.31 ± 0.02	105 ± 52	185 ± 90	0.57 ± 0.12	86 ± 40	124 ± 67	241 ± 130	0.52 ± 0.12	67 ± 38
Cerebral white matter	106 ± 51	187 ± 89	0.56 ± 0.04	134 ± 64	185 ± 90	0.73 ± 0.08	30 ± 14	177 ± 92	241 ± 130	0.74 ± 0.11	30 ± 16

*ROI, Region of interest for each anatomical region. REF, Reference region of interest within the posterior orbit. SU-Ratio, Signal unit ratio refers to signal unit within the particular region of interest (ROI) divided by the unit within the orbita serving as reference (REF). The percentage (%) change refers to the percentage change in signal unit ratio after 24 and 48 h relative to before contrast (Pre)*.

### Ventricular Reflux of Tracer

**Figure 3:**
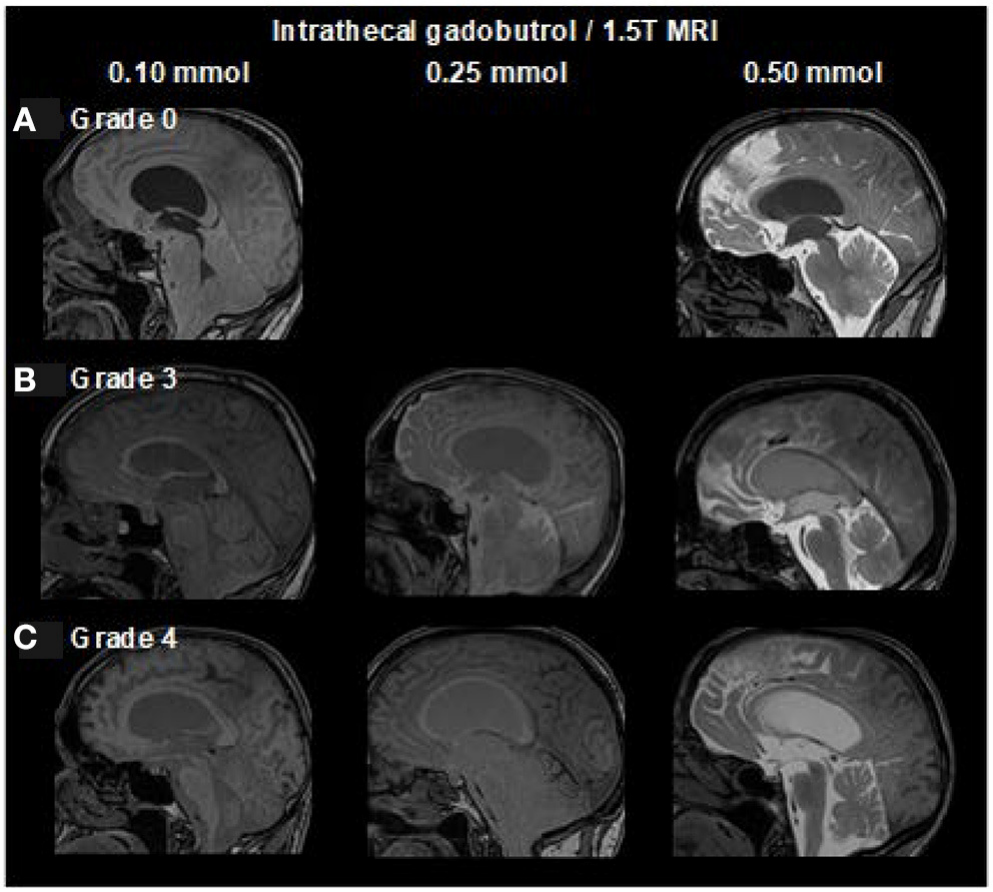
Dose-dependent visualization of ventricular reflux of CSF tracer from sagittal reconstructed T1 weighted images (1.5T MRI) at 24 h after intrathecal gadobutrol in doses of 0.10, 0.25, and 0.50 mmol. Ventricular reflux is categorized into five categories: **(A)** Grade 0: No supra-aqueductal reflux. Grade 1: Sign of supra-aqueductal reflux Day 1. Grade 2: Transient enrichment of lateral ventricles Day 1. **(B)** Grade 3: Lasting enrichment of lateral ventricles Day 2 (not isointense with CSF subarachnoid). **(C)** Grade 4: Lasting enrichment of lateral ventricles Day 2 (isointense with CSF subarachnoid). At 1.5T, MRI was not obtained post-contrast at Day 1, which allowed for scoring of grades 0, 3, and 4 only. In iNPH, we only consider grades 3 to 4 Day 2 at 24 h as abnormal (18).

**Figure 4:**
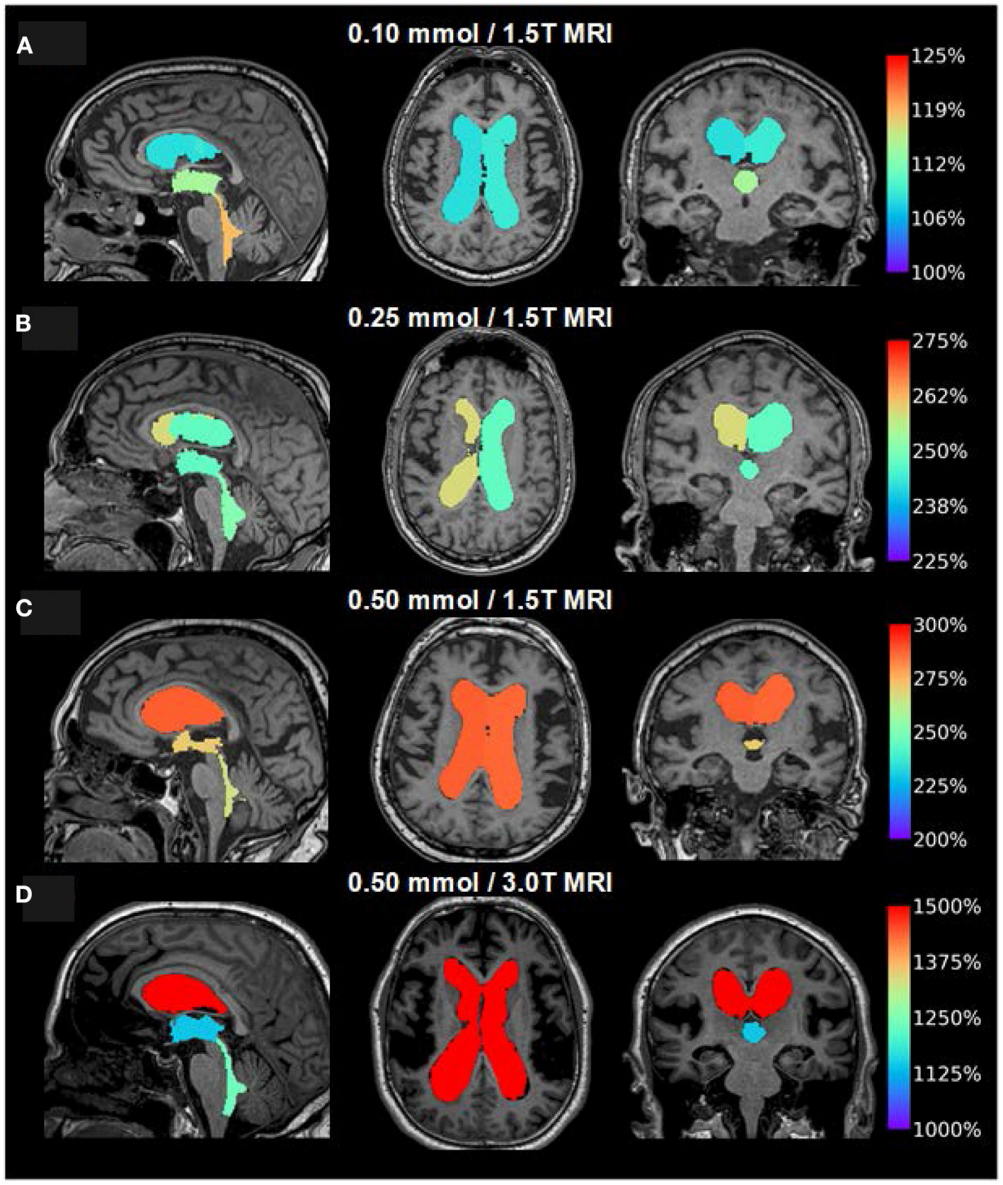
Visualization of dose-dependent tracer enrichment at group level 24 h after intrathecal gadobutrol in the doses **(A)** 0.10 mmol (1.5T MRI; *n* = 18), **(B)** 0.25 mmol (1.5T MRI; *n* = 25), **(C)** 0.50 mmol (1.5T MRI; *n* = 19), and **(D)** 0.50 mmol (3T MRI; *n* = 33). The percentage change in a normalized T1 signal is indicated on the color bar to the right. It should be noted that the scale bar to the right differs for figures **(A–D)**.

**Figure 5:**
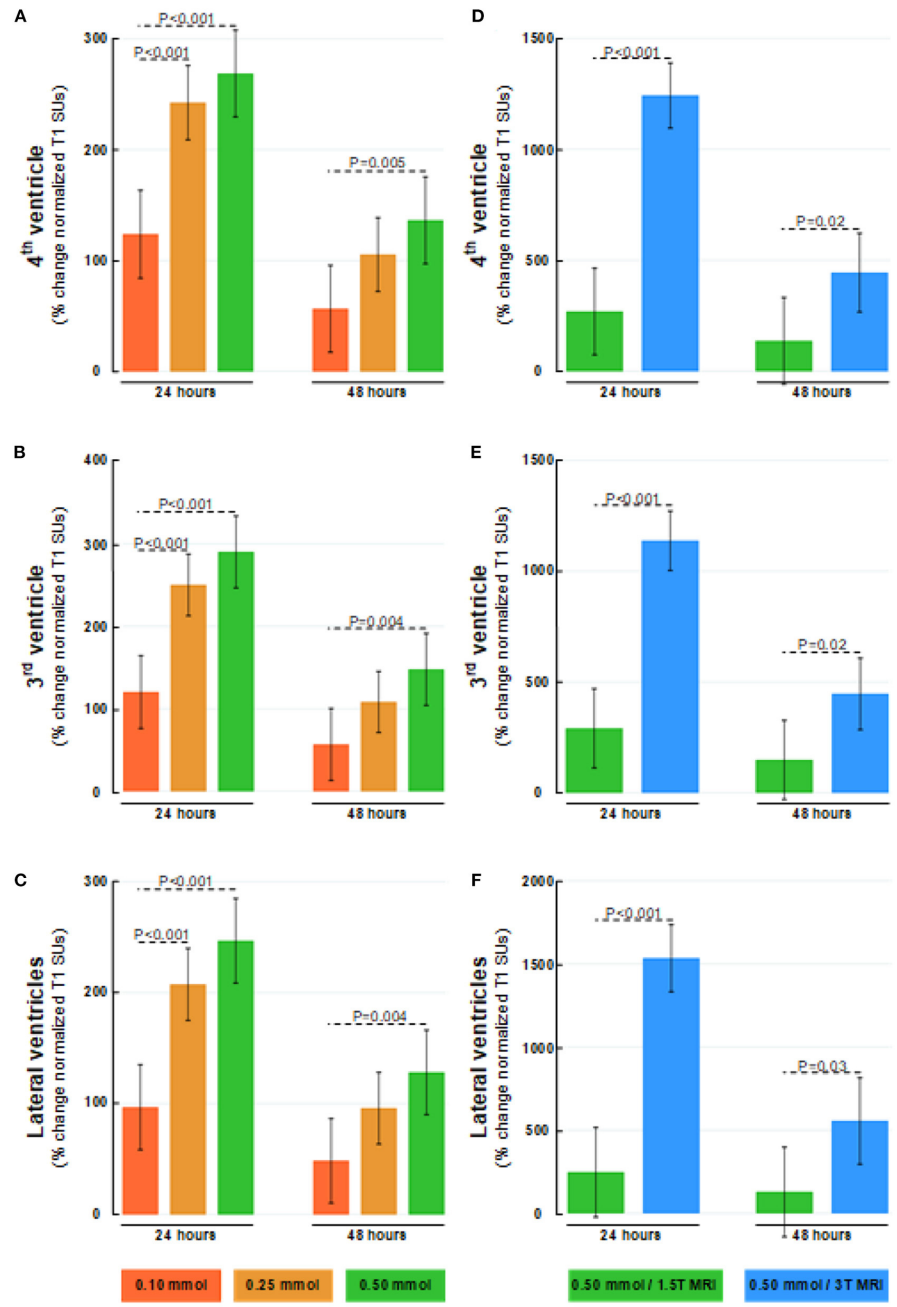
Dose-dependent percentage changes in normalized T1 signal (1.5T MRI scanner) after 24 and 48 h are shown for **(A)** 4th ventricle, **(B)** 3rd ventricle, and **(C)** lateral ventricles following intrathecal gadobutrol in doses of 0.10 (red bars), 0.25 (orange bars), and 0.50 mmol (green bars). The percentage change in normalized T1 signal at 24 and 48 hours after intrathecal gadobutrol (0.50 mmol) within **(D)** 4th ventricle, **(E)** 3rd ventricle, and **(F)** lateral ventricles are shown for 1.5T (green bars) and 3T MRI scanners (blue bars). The bars show mean and 95% CIs. Differences between groups were determined by mixed model analysis.

The grading of ventricular reflux of CSF tracer differed somewhat between groups receiving either 0.10 or 0.25 mmol (Table 2). How differences in ventricular reflux at 24 h are visualized for different doses of intrathecal gadobutrol are illustrated in Figure 3. The percentage increase in normalized T1 signal within ventricles at 24 h at the group level is shown in Figure 4; information about signal units and signal unit ratios are presented in Table 3. As further demonstrated in Figures 5A–C, there was a dose-dependent change in normalized T1 signal within ventricles at 24 h. Notably, all three doses of 0.10, 0.25, and 0.5 mmol were adequate for the visualization of ventricular reflux grade at 24 h (Figures 5A–C). The ventricular tracer enrichment differed markedly between the 1.5T and 3T MRI scanners (Figures 5D–F). Hence, the visualization of ventricular reflux assessment is affected by both dose and magnetic field strength, but the information about reflux grade is maintained by an intrathecal dose of 0.10 mmol gadobutrol.

**Figure 6:**
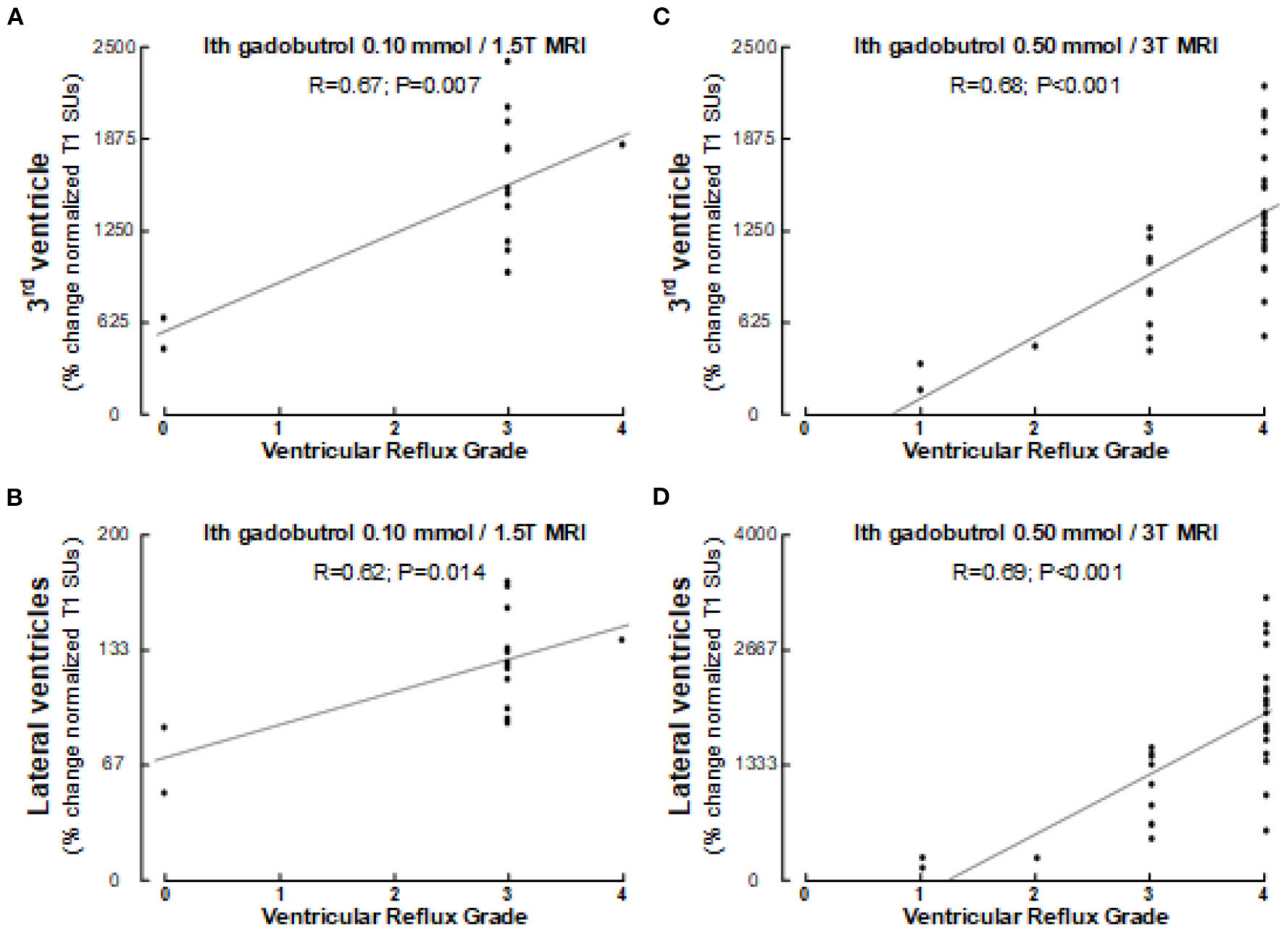
Association between ventricular reflux grade and tracer enrichment 24 h after intrathecal gadobutrol.1 mmol (1.5T MRI) within **(A)** 3rd ventricle and **(B)** lateral ventricles, and between ventricular reflux grade and tracer enrichment 24 h after intrathecal gadobutrol 0.5 mmol (3T MRI) within **(C)** 3rd ventricle and **(D)** lateral ventricles. Pearson correlations with significance levels are shown. Of note, Pearson and Spearman's correlations were similar.

There was a close association between ventricular reflux grade and tracer enrichment within ventricles for intrathecal gadobutrol in doses of 0.10 or 0.50 mmol (Figure 6).

Furthermore, it should be noted that more pronounced ventricular reflux was accompanied by reduced callosal angle (ventricular reflux scores 0–2 vs. 3–4: 95 ± 40° vs. 67 ± 17°; *P* = 0.001; independent samples *t*-test).

### Tracer Enrichment Within the Brain Parenchyma

**Figure 7:**
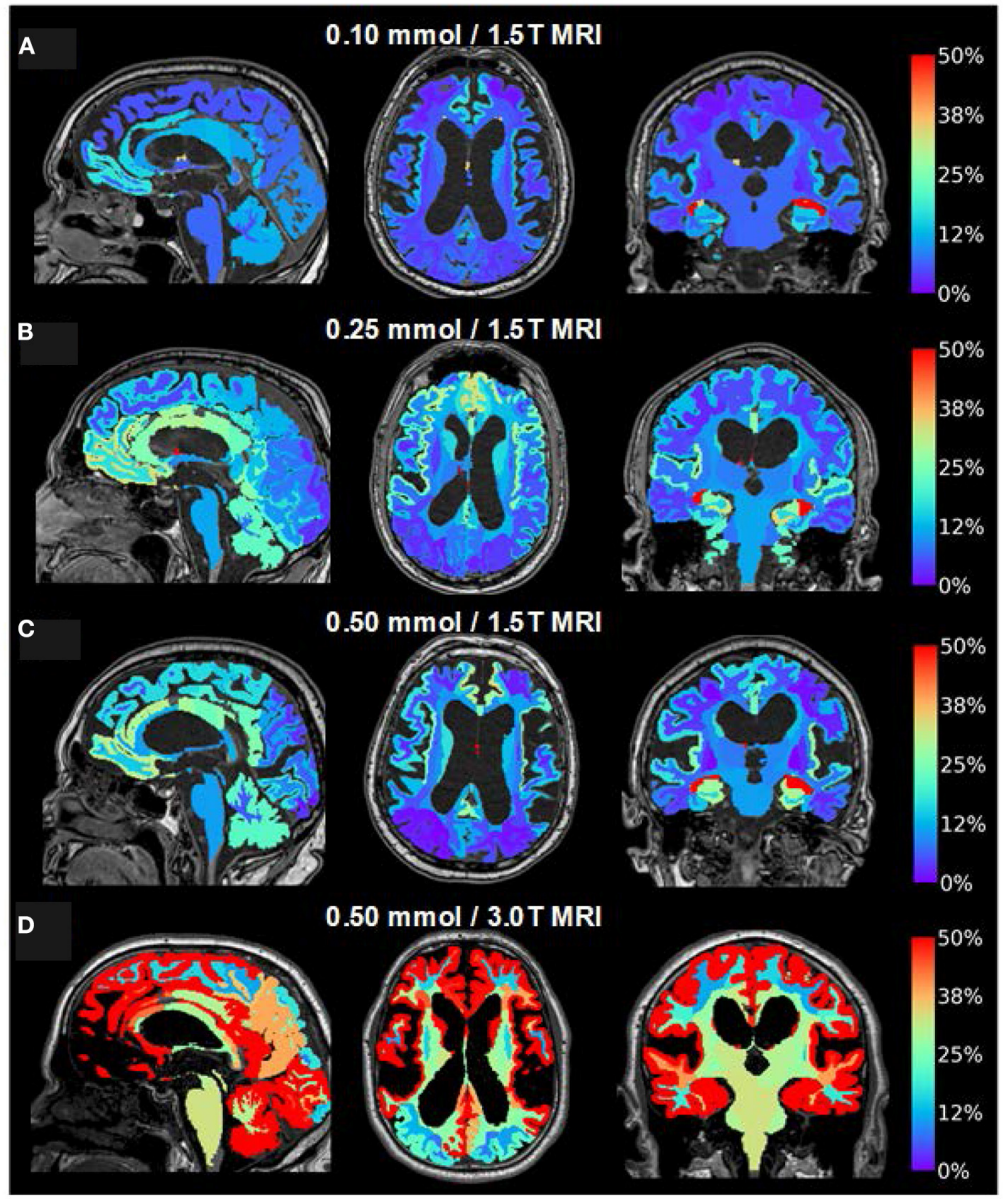
Visualization of dose-dependent brain-wide tracer enrichment at group level 24 h after intrathecal gadobutrol in the doses **(A)** 0.10 mmol (1.5T MRI; *n* = 18), **(B)** 0.25 mmol (1.5T MRI; *n* = 25), **(C)** 0.50 mmol (1.5T MRI; *n* = 19), and **(D)** 0.50 mmol (3T MRI; *n* = 33). The percentage change in the normalized T1 signal is indicated on the color bar to the right.

**Figure 8:**
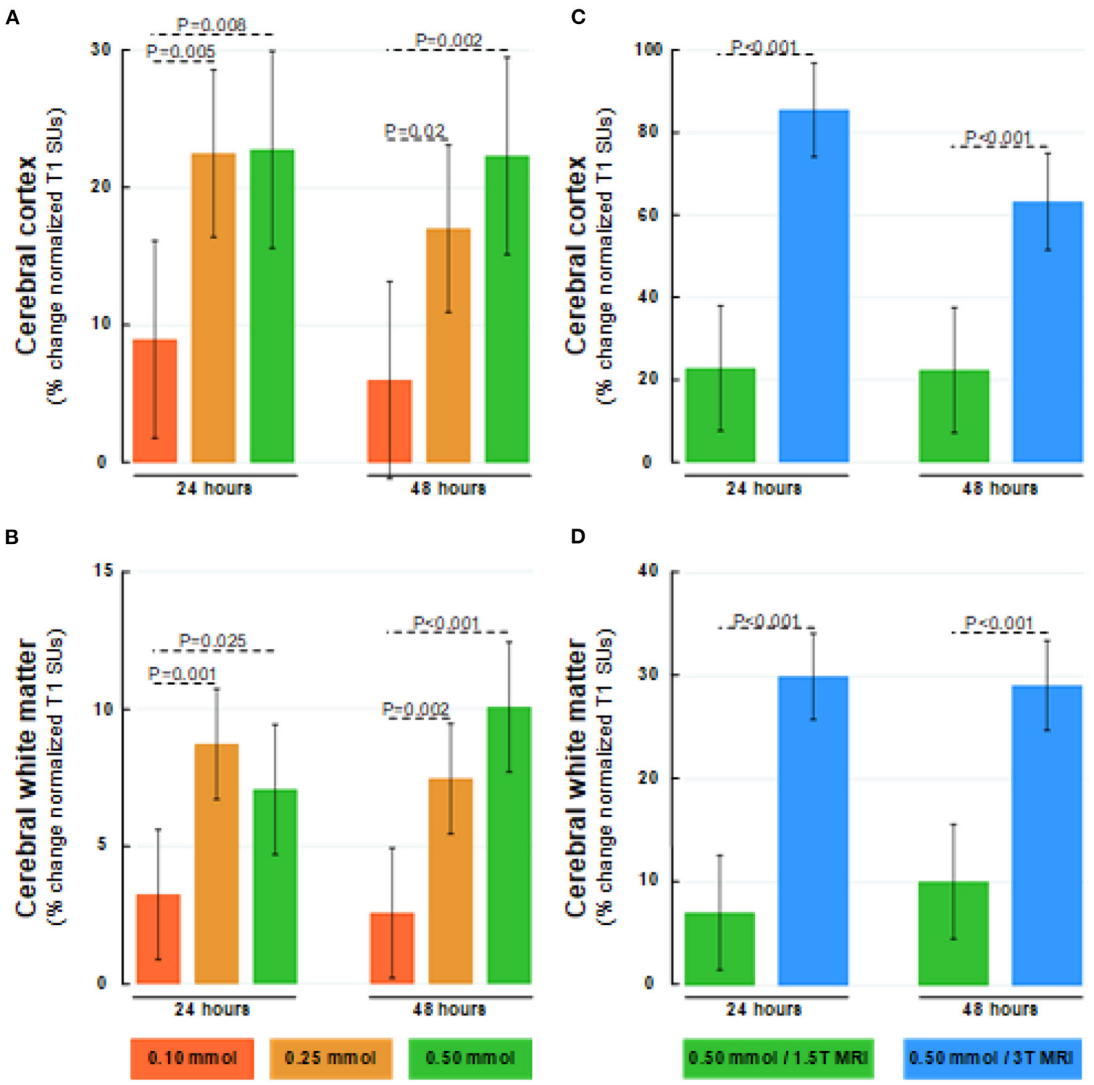
Dose-dependent percentage changes in normalized T1 signal (1.5T MRI scanner) after 24 and 48 h within **(A)** cerebral cortex (gray matter), and **(B)** cerebral white matter after intrathecal gadobutrol in doses of 0.10 mmol (red bars), 0.25 mmol (orange bars), and 0.50 mmol (green bars). The percentage change in normalized T1 signal at 24 and 48 h after intrathecal gadobutrol (0.50 mmol) within **(C)** cerebral cortex (gray matter), and **(D)** cerebral white matter are shown for 1.5T (green bars) and 3T MRI scanners (blue bars). The bars show mean and 95% CIs. Differences between groups were determined by mixed model analysis.

As illustrated in Figure 7, the CSF tracer enrichment within the brain parenchyma, indicative of glymphatic enhancement, depended on the dose of intrathecal gadobutrol, as well as on the application of either 1.5T or 3T MRI. Intrathecal gadobutrol in a dose 0.10 mmol gave significantly less change in normalized T1 signal within the cerebral cortex (Figure 8A) and cerebral white matter (Figure 8B). See also Table 3. An intrathecal dose of 0.10 mmol gadobutrol gave an average increase in normalized T1 signals below 10% in the cerebral cortex (Figure 8A) and below 5% in subcortical white matter (Figure 8B), which we deemed insufficient for assessment of glymphatic enhancement. After intrathecal gadobutrol in a dose of 0.50 mmol, the 1.5T MRI scanner gave markedly lower tracer enrichment in the cerebral cortex (Figure 8C) and cerebral white matter (Figure 8D) than the 3T MRI scanner. The same results were seen within various brain sub-regions, as further detailed in Table 4.

**Table 4:** Dose-dependent change T1 signal units and normalized T1 within different brain regions after 24 h.

	**0.10 mmol/1.5T MRI**										
	**Pre**			**24 h**				**48 h**			
**Anatomical region**	**ROI**	**REF**	**Ratio**	**ROI**	**REF**	**Ratio**	**%Change**	**ROI**	**REF**	**Ratio**	**%Change**
Frontal cortex (GM)	214 ± 27	386 ± 59	0.56 ± 0.05	233 ± 30	385 ± 56	0.61 ± 0.06	10 ± 8	232 ± 30	397 ± 61	0.59 ± 0.04	6 ± 5
Frontal cortex (WM)	273 ± 35	386 ± 59	0.71 ± 0.06	280 ± 32	385 ± 56	0.73 ± 0.06	3 ± 4	287 ± 36	397 ± 61	0.73 ± 0.05	2 ± 3
Temporal cortex (GM)	228 ± 27	386 ± 59	0.59 ± 0.04	251 ± 32	385 ± 56	0.66 ± 0.06	11 ± 7	251 ± 34	397 ± 61	0.63 ± 0.05	7 ± 4
Temporal cortex (WM)	292 ± 38	386 ± 59	0.76 ± 0.05	303 ± 37	385 ± 56	0.79 ± 0.06	5 ± 4	312 ± 44	397 ± 61	0.79 ± 0.06	4 ± 3
Parietal cortex (GM)	221 ± 26	386 ± 59	0.58 ± 0.05	237 ± 31	385 ± 56	0.62 ± 0.05	8 ± 7	240 ± 34	397 ± 61	0.61 ± 0.06	6 ± 5
Parietal cortex (WM)	281 ± 34	386 ± 59	0.73 ± 0.06	287 ± 36	385 ± 56	0.75 ± 0.05	3 ± 4	294 ± 39	397 ± 61	0.75 ± 0.06	2 ± 3
Occipital cortex (GM)	231 ± 28	386 ± 59	0.60 ± 0.05	251 ± 35	385 ± 56	0.66 ± 0.05	9 ± 7	253 ± 37	397 ± 61	0.64 ± 0.05	7 ± 5
Occipital cortex (WM)	292 ± 35	386 ± 59	0.76 ± 0.06	302 ± 38	385 ± 56	0.79 ± 0.06	4 ± 5	309 ± 44	397 ± 61	0.78 ± 0.07	3 ± 4
Cerebellar cortex (GM)	238 ± 28	386 ± 59	0.62 ± 0.05	266 ± 32	385 ± 56	0.70 ± 0.07	13 ± 6	264 ± 38	397 ± 61	0.67 ± 0.06	8 ± 5
Cerebellar cortex (WM)	293 ± 35	386 ± 59	0.76 ± 0.06	301 ± 34	385 ± 56	0.79 ± 0.07	4 ± 5	310 ± 43	397 ± 61	0.78 ± 0.07	3 ± 4
Brainstem	281 ± 39	386 ± 59	0.73 ± 0.06	292 ± 40	385 ± 56	0.76 ± 0.08	5 ± 8	299 ± 46	397 ± 61	0.75 ± 0.06	4 ± 6
Basal ganglia	262 ± 31	386 ± 59	0.68 ± 0.06	273 ± 33	385 ± 56	0.71 ± 0.06	5 ± 4	278 ± 39	397 ± 61	0.70 ± 0.06	3 ± 3
Limbic structures	255 ± 31	386 ± 59	0.66 ± 0.05	282 ± 36	385 ± 56	0.74 ± 0.06	12 ± 5	277 ± 39	397 ± 61	0.70 ± 0.05	6 ± 3
	**0.25 mmol/1.5T MRI**										
Frontal cortex (GM)	194 ± 30	365 ± 44	0.53 ± 0.07	251 ± 27	382 ± 49	0.66 ± 0.08	26 ± 21	241 ± 27	389 ± 56	0.63 ± 0.07	19 ± 18
Frontal cortex (WM)	252 ± 29	365 ± 44	0.69 ± 0.05	287 ± 26	382 ± 49	0.76 ± 0.06	9 ± 5	290 ± 31	389 ± 56	0.75 ± 0.06	8 ± 6
Temporal cortex (GM)	208 ± 30	365 ± 44	0.57 ± 0.06	266 ± 29	382 ± 49	0.70 ± 0.08	24 ± 18	254 ± 28	389 ± 56	0.66 ± 0.06	16 ± 13
Temporal cortex (WM)	269 ± 32	365 ± 44	0.74 ± 0.06	309 ± 31	382 ± 49	0.82 ± 0.07	10 ± 6	309 ± 36	389 ± 56	0.80 ± 0.06	8 ± 5
Parietal cortex (GM)	199 ± 31	365 ± 44	0.55 ± 0.07	243 ± 33	382 ± 49	0.64 ± 0.08	18 ± 17	243 ± 31	389 ± 56	0.63 ± 0.08	16 ± 17
Parietal cortex (WM)	259 ± 28	365 ± 44	0.71 ± 0.05	289 ± 28	382 ± 49	0.76 ± 0.06	7 ± 5	292 ± 31	389 ± 56	0.76 ± 0.06	6 ± 6
Occipital cortex (GM)	210 ± 31	365 ± 44	0.58 ± 0.06	260 ± 33	382 ± 49	0.68 ± 0.08	19 ± 15	253 ± 30	389 ± 56	0.66 ± 0.06	14 ± 11
Occipital cortex (WM)	272 ± 30	365 ± 44	0.75 ± 0.05	308 ± 33	382 ± 49	0.81 ± 0.06	9 ± 6	305 ± 35	389 ± 56	0.79 ± 0.05	8 ± 5
Cerebellar cortex (GM)	224 ± 24	365 ± 44	0.62 ± 0.05	291 ± 32	382 ± 49	0.77 ± 0.09	25 ± 11	271 ± 30	389 ± 56	0.70 ± 0.06	14 ± 6
Cerebellar cortex (WM)	275 ± 30	365 ± 44	0.76 ± 0.05	311 ± 31	382 ± 49	0.82 ± 0.07	8 ± 6	308 ± 36	389 ± 56	0.80 ± 0.06	5 ± 4
Brainstem	267 ± 31	365 ± 44	0.74 ± 0.06	311 ± 33	382 ± 49	0.82 ± 0.08	11 ± 6	300 ± 38	389 ± 56	0.78 ± 0.07	6 ± 4
Basal ganglia	248 ± 27	365 ± 44	0.68 ± 0.04	287 ± 26	382 ± 49	0.76 ± 0.06	12 ± 6	282 ± 32	389 ± 56	0.73 ± 0.05	7 ± 4
Limbic structures	237 ± 27	365 ± 44	0.65 ± 0.05	303 ± 28	382 ± 49	0.80 ± 0.08	24 ± 11	280 ± 31	389 ± 56	0.72 ± 0.05	12 ± 6
	**0.50 mmol/1.5T MRI**										
Frontal cortex (GM)	207 ± 26	382 ± 42	0.54 ± 0.05	263 ± 43	389 ± 42	0.68 ± 0.09	26 ± 26	267 ± 35	403 ± 43	0.66 ± 0.07	24 ± 21
Frontal cortex (WM)	268 ± 29	382 ± 42	0.70 ± 0.05	294 ± 29	389 ± 42	0.76 ± 0.06	8 ± 9	312 ± 32	403 ± 43	0.78 ± 0.05	11 ± 9
Temporal cortex (GM)	221 ± 27	382 ± 42	0.58 ± 0.05	281 ± 45	389 ± 42	0.72 ± 0.10	26 ± 26	283 ± 33	403 ± 43	0.71 ± 0.07	23 ± 18
Temporal cortex (WM)	287 ± 32	382 ± 42	0.75 ± 0.05	318 ± 33	389 ± 42	0.82 ± 0.06	9 ± 9	335 ± 32	403 ± 43	0.84 ± 0.06	12 ± 9
Parietal cortex (GM)	213 ± 30	382 ± 42	0.56 ± 0.06	251 ± 33	389 ± 42	0.65 ± 0.08	17 ± 23	266 ± 32	403 ± 43	0.66 ± 0.07	20 ± 21
Parietal cortex (WM)	274 ± 30	382 ± 42	0.72 ± 0.05	292 ± 24	389 ± 42	0.75 ± 0.05	4 ± 6	311 ± 29	403 ± 43	0.78 ± 0.05	8 ± 7
Occipital cortex (GM)	226 ± 32	382 ± 42	0.59 ± 0.06	269 ± 36	389 ± 42	0.69 ± 0.08	17 ± 17	281 ± 33	403 ± 43	0.70 ± 0.07	19 ± 17
Occipital cortex (WM)	289 ± 32	382 ± 42	0.76 ± 0.06	312 ± 31	389 ± 42	0.81 ± 0.06	6 ± 7	329 ± 28	403 ± 43	0.82 ± 0.06	8 ± 8
Cerebellar cortex (GM)	236 ± 23	382 ± 42	0.62 ± 0.04	307 ± 52	389 ± 42	0.79 ± 0.12	28 ± 21	300 ± 35	403 ± 43	0.75 ± 0.09	22 ± 16
Cerebellar cortex (WM)	291 ± 28	382 ± 42	0.76 ± 0.05	318 ± 30	389 ± 42	0.82 ± 0.06	7 ± 8	332 ± 29	403 ± 43	0.83 ± 0.06	9 ± 8
Brainstem	282 ± 28	382 ± 42	0.74 ± 0.05	320 ± 35	389 ± 42	0.83 ± 0.07	12 ± 10	325 ± 33	403 ± 43	0.81 ± 0.07	9 ± 7
Basal ganglia	260 ± 23	382 ± 42	0.68 ± 0.04	291 ± 25	389 ± 42	0.75 ± 0.06	11 ± 10	300 ± 25	403 ± 43	0.75 ± 0.05	10 ± 8
Limbic structures	252 ± 25	382 ± 42	0.66 ± 0.04	320 ± 46	389 ± 42	0.83 ± 0.11	26 ± 19	309 ± 32	403 ± 43	0.77 ± 0.07	17 ± 12
	**0.50 mmol/3T MRI**										
Frontal cortex (GM)	56 ± 31	187 ± 89	0.29 ± 0.02	104 ± 52	185 ± 90	0.56 ± 0.12	96 ± 44	121 ± 66	241 ± 130	0.51 ± 0.12	74 ± 42
Frontal cortex (WM)	102 ± 50	187 ± 89	0.54 ± 0.04	129 ± 62	185 ± 90	0.70 ± 0.08	30 ± 14	173 ± 92	241 ± 130	0.73 ± 0.11	32 ± 17
Temporal cortex (GM)	57 ± 28	187 ± 89	0.30 ± 0.02	109 ± 52	185 ± 90	0.60 ± 0.13	97 ± 41	120 ± 63	241 ± 130	0.51 ± 0.13	71 ± 38
Temporal cortex (WM)	105 ± 47	187 ± 89	0.56 ± 0.04	142 ± 65	185 ± 90	0.78 ± 0.10	39 ± 17	179 ± 91	241 ± 130	0.76 ± 0.12	35 ± 18
Parietal cortex (GM)	64 ± 36	187 ± 89	0.34 ± 0.03	102 ± 50	185 ± 90	0.55 ± 0.12	65 ± 38	128 ± 69	241 ± 130	0.54 ± 0.11	60 ± 35
Parietal cortex (WM)	110 ± 56	187 ± 89	0.59 ± 0.04	133 ± 64	185 ± 90	0.72 ± 0.08	23 ± 13	178 ± 92	241 ± 130	0.75 ± 0.09	25 ± 15
Occipital cortex (GM)	68 ± 38	187 ± 89	0.35 ± 0.03	108 ± 55	185 ± 90	0.58 ± 0.12	63 ± 35	132 ± 74	241 ± 130	0.55 ± 0.11	53 ± 32
Occipital cortex (WM)	112 ± 57	187 ± 89	0.59 ± 0.04	140 ± 69	185 ± 90	0.75 ± 0.09	27 ± 15	180 ± 96	241 ± 130	0.75 ± 0.10	23 ± 14
Cerebellar cortex (GM)	67 ± 32	187 ± 89	0.36 ± 0.03	129 ± 63	185 ± 90	0.71 ± 0.13	98 ± 35	139 ± 70	241 ± 130	0.59 ± 0.11	64 ± 25
Cerebellar cortex (WM)	113 ± 52	187 ± 89	0.61 ± 0.04	148 ± 74	185 ± 90	0.80 ± 0.08	32 ± 13	182 ± 96	241 ± 130	0.76 ± 0.09	25 ± 10
Brainstem	118 ± 57	187 ± 89	0.63 ± 0.04	156 ± 76	185 ± 90	0.84 ± 0.07	34 ± 12	188 ± 101	241 ± 130	0.78 ± 0.09	23 ± 11
Basal ganglia	94 ± 47	187 ± 89	0.50 ± 0.04	126 ± 61	185 ± 90	0.68 ± 0.07	42 ± 18	156 ± 83	241 ± 130	0.65 ± 0.08	29 ± 15
Limbic structures	86 ± 41	187 ± 89	0.46 ± 0.03	148 ± 69	185 ± 90	0.81 ± 0.12	82 ± 27	158 ± 81	241 ± 130	0.67 ± 0.11	48 ± 23

*ROI, Region of interest for each anatomical region. REF, Reference region of interest within the posterior orbit. SU-Ratio, Signal unit ratio refers to signal unit within the particular region of interest (ROI) divided by the signal unit within the orbita serving as reference (REF). The percentage (%) change refers to the percentage change in signal unit ratio after 24 and 48 h relative to before contrast (Pre)*.

As shown in Table 5, there was a highly significant positive correlation between tracer enrichment within the CSF of subarachnoid spaces and tracer enrichment within the brain parenchyma, though correlations were strongest for intrathecal gadobutrol in a dose of 0.5 mmol. Furthermore, the location of measurements matters, with the strongest correlations between CSF tracer at vertex and enrichment in the cerebral cortex and the strongest correlations between CSF tracer at cisterna magna and enrichment within the entorhinal cortex.

**Table 5:** Correlations between tracer enrichment within subarachnoid CSF spaces and brain parenchyma.

	**24 h after ith gadobutrol**			**48 h after ith gadobutrol**		
	**CSF cisterna magna**	**CSF vertex**	**CSF velum interpositum**	**CSF cisterna magna**	**CSF vertex**	**CSF velum interpositum**
**1.5T MRI**						
Ith gadobutrol 0.10 mmol						
Cerebral cortex (GM)	*R =* 0.32; ns	*R =* 0.65; *P =* 0.009	*R =* 0.06; ns	*R =* 0.33; ns	*R =* 0.53; *P =* 0.041	*R =* −0.13; ns
Cerebral white matter	*R =* 0.22; ns	*R =* 0.47; ns	*R =* 0.10; ns	*R =* 0.30; ns	*R =* 0.50; ns	*R =* −0.26; ns
Entorhinal cortex (GM)	*R =* 0.54; *P =* 0.040	*R =* 0.55; *P =* 0.034	*R =* −0.03; ns	*R =* 0.51; *P =* 0.054	*R =* 0.44; ns	*R =* 0.07; ns
Entorhinal cortex (WM)	*R =* 0.44; ns	*R =* 0.47; ns	*R =* 0.04; ns	*R =* 0.62; *P =* 0.013	*R =* 0.39; ns	*R =* −0.09; ns
Ith gadobutrol 0.25 mmol						
Cerebral cortex (GM)	*R =* 0.38; ns	*R =* 0.28; ns	*R =* 0.82; *P < * 0.001	*R =* 0.42; *P =* 0.041	*R =* 0.55; *P =* 0.005	*R =* 0.24; ns
Cerebral white matter	*R =* 0.43; *P =* 0.036	*R =* 0.49; *P =* 0.015	–	*R =* 0.27; ns	*R =* 0.58; *P =* 0.003	*R =* 0.36; ns
Entorhinal cortex (GM)	*R =* 0.61; *P =* 0.002	*R =* 0.08; ns	*R =* 0.30; ns	*R =* 0.59; *P =* 0.002	*R =* 0.43; *P =* 0.035	*R =* 0.33; ns
Entorhinal cortex (WM)	*R =* 0.67; *P < * 0.001	*R =* 0.25; ns	*R =* 0.43; *P =* 0.035	*R =* 0.46; *P =* 0.025	*R =* 0.35; ns	*R =* 0.36; ns
Ith gadobutrol 0.50 mmol						
Cerebral cortex (GM)	*R =* 0.60; *P =* 0.015	*R =* 0.75; *P =* 0.001	*R =* 0.52; *P =* 0.029	*R =* 0.73; *P =* 0.001	*R =* 0.63; *P =* 0.009	*R =* 0.60; *P =* 0.008
Cerebral white matter	*R =* 0.63; *P =* 0.009	*R =* 0.58; *P =* 0.02	*R =* 0.78; *P < * 0.001	*R =* 0.76; *P =* 0.001	*R =* 0.50; *P =* 0.049	*R =* 0.81; *P < * 0.001
Entorhinal cortex (GM)	*R =* 0.81; *P < * 0.001	*R =* 0.59; *P =* 0.016	*R =* 0.78; *P < * 0.001	*R =* 0.90; *P < * 0.001	*R =* 0.54; *P =* 0.030	*R =* 0.88; *P < * 0.001
Entorhinal cortex (WM)	*R =* 0.73; *P =* 0.001	*R =* 0.55; *P =* 0.026	*R =* 0.82; *P < * 0.001	*R =* 0.78; *P < * 0.001	*R =* 0.47; ns	*R =* 0.88; *P < * 0.001
**3T MRI**
Ith gadobutrol 0.50 mmol						
Cerebral cortex (GM)	*R =* 0.53; *P =* 0.002	*R =* 0.67; *P < * 0.001	*R =* 0.38; *P =* 0.032	*R =* 0.80; *P =* 0.006	*R =* 0.70; *P =* 0.023	*R =* 0.44; ns
Cerebral white matter	*R =* 0.56; *P =* 0.001	*R =* 0.61; *P < * 0.001	*R =* 0.40; *P =* 0.025	*R =* 0.77; *P =* 0.009	*R =* 0.73; *P =* 0.017	*R =* 0.40; ns
Entorhinal cortex (GM)	*R =* 0.74; *P < * 0.001	*R =* 0.49; *P =* 0.005	*R =* 0.48; *P =* 0.005	*R =* 0.88; *P =* 0.001	*R =* 0.60; ns	*R =* 0.57; ns
Entorhinal cortex (WM)	*R =* 0.70; *P < * 0.001	*R =* 0.50; *P =* 0.004	*R =* 0.51; *P =* 0.003	*R =* 0.94; *P < * 0.001	*R =* 0.69; *P =* 0.026	*R =* 0.46; ns

*Pearson correlations were determined from percentage change in tracer enrichment (normalized T1 signal units) after 24 and 48 h as Pearson correlation coefficients with the significance level*.

**Figure 9:**
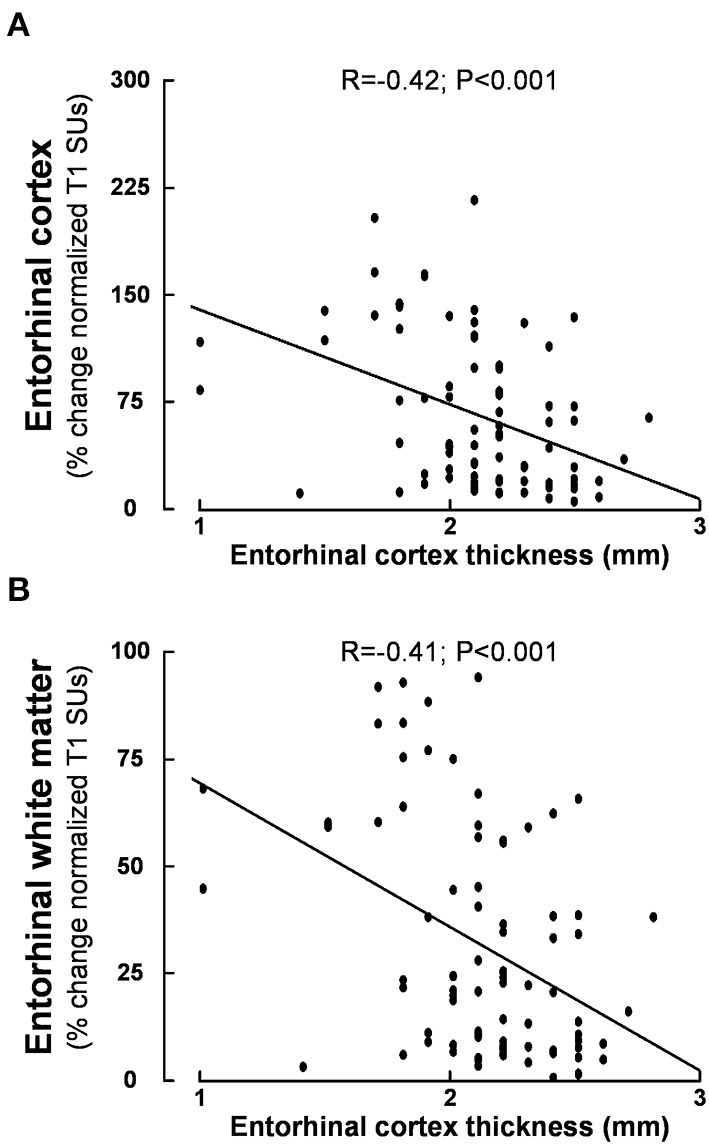
Association between entorhinal cortex thickness and tracer enrichment at 24 h within **(A)** entorhinal cortex and **(B)** entorhinal white matter. Higher values of normalized T1 signal units are indicative of reduced parenchymal clearance of the tracer. Pearson correlations with significance levels are shown. Of note, Pearson and Spearman's correlations were similar. This plot is based on 84 patients.

Considering the entire material, we also note a significant relationship between entorhinal cortex (ERC) thickness and tracer enrichment within entorhinal cortex gray matter (Figure 9A) and entorhinal cortex white matter (Figure 9B). Thereby, reduced clearance of tracer from entorhinal cortex shown as higher tracer levels at 24 h was accompanied by the reduced thickness of the entorhinal cortex.

## Discussion

This study provides new insights about the utility of intrathecal gadobutrol to assess MRI biomarkers of CSF dynamics and glymphatic enhancement, where a reduction of dose from 0.50 to 0.25 mmol maintained necessary diagnostic information. We deemed a dose of 0.10 mmol insufficient because of too low tracer enrichment in CSF at the vertex and too low enrichment in the cerebral cortex and subcortical white matter. In iNPH, we found significant reflux of tracer toward ventricles (grades 3–4, indicative of marked ventricular re-direction of CSF flow), and a strong association between clearance of tracer from CSF and brain parenchyma, suggesting brain molecular clearance is dependent on CSF clearance. The modest tracer enrichment beneath the skull vertex indicates a minor role of arachnoid granulations in CSF efflux.

In previous studies, we have applied intrathecal gadobutrol in a dose of 0.5 mmol, which has been found safe (30, 31). A systematic review concluded that no severe complications have been shown for gadolinium-based contrast agents in doses of 1.0 mmol and lower, though toxic effects have been shown in doses above 1.0 mmol (28). The present study provides evidence that the diagnostic imaging information of intrathecal gadobutrol is maintained at 0.25 mmol, while a dose of 0.10 mmol seems too low at 1.5T. Moreover, 3T MRI seems preferable above 1.5T MRI, while the latter is sufficient for the biomarkers in question with a dose of 0.25 mmol. Intrathecal MRI contrast agents are presently used off-label, primarily because of potential neurotoxicity and concerns about deposition within the brain (44). However, the risk of deposition within the brain of gadobutrol when given in intrathecal doses of 0.25 or 0.50 mmol seems minor 0. After 4 weeks, we have not found changes in normalized T1 signals in any brain regions (27). After routinely intravenous administration, there is also the passage of contrast to the CSF in humans (45–47) as previously shown in animals (48). Gadobutrol is approved for intravenous use in dosage of 0.1–0.3 mmol/kg, which in a 80 kg adult represents 8–24 mmol body dose, i.e., 16–60 times higher than intrathecal doses of 0.25 and 0.50 mmol, respectively. Therefore, we consider that intrathecal gadobutrol in doses 0.25–0.50 mmol has an acceptable risk profile while the benefit is substantial, given the opportunity to retrieve unique information about disturbed CSF homeostasis. In our opinion, the therapeutic index (i.e., risk-benefit ratio) of intrathecal gadobutrol is acceptable, justifying its clinical application.

In this study, the increasing dose from 0.25 to 0.50 mmol at 1.5T provided only a modest signal increase in CSF spaces and brain tissue (Figures 2, 5, 8). At this magnetic field strength, it, therefore, seems reasonable to avoid doses higher than 0.25 mmol, while 0.10 mmol was deemed insufficient. It, therefore, seems that an intrathecal dose of 0.25 mmol is close to ideal for 1.5T. The effect on percentage signal increase in CSF and brain tissue by increasing the magnetic field strength was, on the other side, much larger than the effect of increasing the contrast dose. We can therefore not rule out that 0.10 mmol may be sufficient at field strengths higher than 1.5T, and this could be explored in later studies. While the intrinsic signal-to-noise ratio (SNR) in MRI increases with field strength, signal unit change will also depend on the applied TR and TE, and the impact on using 3T at 0.1 mmol will therefore also depend on sequence parameters (49). It should also be noted that T1 signal increase in the MRIs is not necessarily proportional with the concentration of contrast agent, therefore the percentage change in normalized signal units is in this study used with the intention to illustrate by numbers how effects appear visually to a reader of MR images. To further increase sensitivity for the detection of contrast agents within the CSF spaces, other sequences might in future studies show to be of benefit, for instance, post-contrast FLAIR or T1 with blood suppression (“black blood”). Whether these techniques can maintain sufficient sensitivity for detection of enhancement within the brain remains to be seen.

We have previously shown that ventricular reflux of tracer characterizes iNPH disease (18). This study extends our previous observations showing ventricular reflux grades 3–4 in about 9/10 patients with iNPH. Estimation of tracer enrichment within ventricles using FreeSurfer software showed that tracer enrichment closely follows the categorical grading of reflux. Phase-contrast MRI supports the net retrograde aqueductal flow of CSF in iNPH (17, 50, 51). Others also reported net retrograde CSF flow within the cerebral aqueduct in patients with communicating hydrocephalus (50, 52–56). On the contrary, other disease categories of CSF disturbance, e.g., idiopathic intracranial hypertension and spontaneous intracranial hypotension or brain cysts, demonstrated no ventricular reflux of tracer (17). Likewise, individuals without CSF disturbance showed no ventricular tracer reflux (17), or net retrograde aqueductal flow (51). The ventricular reflux grades 3–4 indicates net CSF flow from fourth to third to lateral ventricles, indicating redirection of CSF flow in iNPH disease. Accordingly, a pressure gradient toward the ventricles enables molecular passage via the cerebral aqueduct into the lateral ventricles and transependymal fluid transport to periventricular white matter. Efflux of CSF also seems to occur *via* the choroid plexus (57). It is of note that reflux grades 3–4 were accompanied by reduced callosal angle as compared with reflux grades 0–2. Hence, in iNPH, the inward pressure gradient, molecular reflux, and need for transependymal transport may underlie the particular ventricular shape characterized by reduced callosal angle and upward movement of the brain along the *z*-axis (58).

Whether reflux grade is predictive for shunt responsiveness was out of scope for this work. We have previously shown that patients with iNPH with reflux grades 3–4 also presented with increased pulsatile ICP during overnight ICP monitoring (18), which is highly predictive for shunt responsiveness in iNPH (5). This patient material only included patients with iNPH. With regard to ventricular reflux, only 6 individuals had reflux grades 0–2, in part since grades 1–2 could not be scored at exams performed at 1.5T due to the imaging routine. However, further studies are needed to address this.

Tracer enrichment at vertex peaked after 48 h for all doses, while enrichment in cerebral ventricles peaked at 24 h. In a previous study (59), we found that the time to peak concentration in blood of intrathecal gadobutrol (0.5 mmol) was 12.1 ± 3.8 h. Molecular egress from CSF to blood is therefore much faster than peak CSF concentration at a vertex. While the traditional view states that arachnoid granulations serve as a major route for CSF efflux (60), the present observations point to a minor role of this efflux route.

The brain-wide enrichment of tracer occurs in the extra-vascular space since the tracer is contained outside the blood vessels because of the blood-brain barrier. We refer to this as glymphatic enhancement since the tracer passes in the perivascular spaces and the interstitial tissue. Tracer enrichment is most pronounced in brain areas nearby large blood vessels, which may indicate a role of the forces created by the pulsatile arteries. The present observations indicate comparable tracer enrichment within brain parenchyma at 24 h for intrathecal gadobutrol in doses of 0.25 and 0.50 mmol, though tracer was far better visualized by 3T than 1.5T MRI, i.e., the effect of increasing magnetic field strength was larger on contrast dependent T1 signal increase.

It is reasonable to hypothesize that the transport of gadobutrol within the CSF and brain compartments mimics the transport of other molecules and metabolic by-products such as amyloid-β and tau. Intrathecal gadobutrol with a molecular weight of about 604 Da does not cross a healthy BBB and distributes in the brain via extra-vascular spaces (61). This contrast agent is highly hydrophilic with an estimated hydraulic diameter of <2 nm (27), enabling it to pass between the perivascular and interstitial space via astrocytic endfeet gaps of about 20 nm. In comparison, amyloid-β isomers and tau are cleared along extra-vascular pathways (22, 62); the outer diameter of amyloid-β oligomers is as well <20 nm (63). While there is no known BBB transporter for tau, some common amyloid-β isoforms have significant clearance over the BBB, even though some of the most toxic amyloid-β isoforms are clear *via* the extravascular pathways (64, 65). Therefore, clearance of gadobutrol from the brain may be a suitable surrogate marker for brain clearance of endogenous metabolites such as toxic amyloid-β isoforms and tau.

Since dementia is an important part of iNPH disease, we have particularly addressed the alterations occurring within the entorhinal cortex. This region in the medial temporal lobe provides a major convergent neuronal input to the hippocampus after receiving direct projections from the neocortex. The entorhinal-hippocampal circuit plays a key role in learning and memories for locations and events (66–68). In Alzheimer's disease, neuronal degeneration within the entorhinal cortex occurs at an early time (69). Numerous studies have provided evidence of thinning of the entorhinal cortex visualized by MRI in mild cognitive impairment and dementia such as early Alzheimer's disease (70–74). Moreover, degeneration and thinning of the entorhinal cortex were accompanied by increased postmortem neurofibrillary tangle burden and amyloid-β (Aβ) load (75). We previously reported that patients with iNPH presented with reduced entorhinal cortex thickness, as compared with references (18). The present data extend previous observations by demonstrating a significant negative correlation between entorhinal cortex thickness and normalized T1 signal units at 24 h within the entorhinal cortex and entorhinal cortex white matter. Accordingly, higher tracer enrichment at 24 h, which is indicative of reduced molecular clearance, was accompanied by thinning of the entorhinal cortex. This supports the idea that reduced clearance of toxic metabolic by-products may be accompanied by neurodegeneration and hence reduced cortical thickness. The present data highlight that there may be some differences when estimating entorhinal cortex thickness from 1.5T and 3T MRI, this is probably an effect of increased SNR at 3T MRI and thereby better ability to discriminate the cortical boundaries.

One important observation supporting our previous findings (24, 26) is that tracer enrichment within the brain, here exemplified by the cerebral cortex and entorhinal cortex, is strongly correlated with tracer enrichment in CSF. The correlation was strongest for nearby CSF and parenchymal regions. Since diffusion may be an important mechanism behind molecular transport in the brain (76), the concentration within the CSF may as well be crucial for molecular brain enrichment from CSF. In patients with iNPH, molecular clearance from subarachnoid spaces is impaired (24); the present data further show that clearance of tracer from the subarachnoid CSF spaces was dose-dependent. Concerning glymphatic function, the role of CSF *per se* has received less attention. For a particular molecule, its glymphatic transport probably is affected by the concentration within the subarachnoid CSF spaces. In this regard, it may be proposed that the meningeal lymphatic vessels play a key role in molecular egress from CSF spaces.

**Figure 10:**
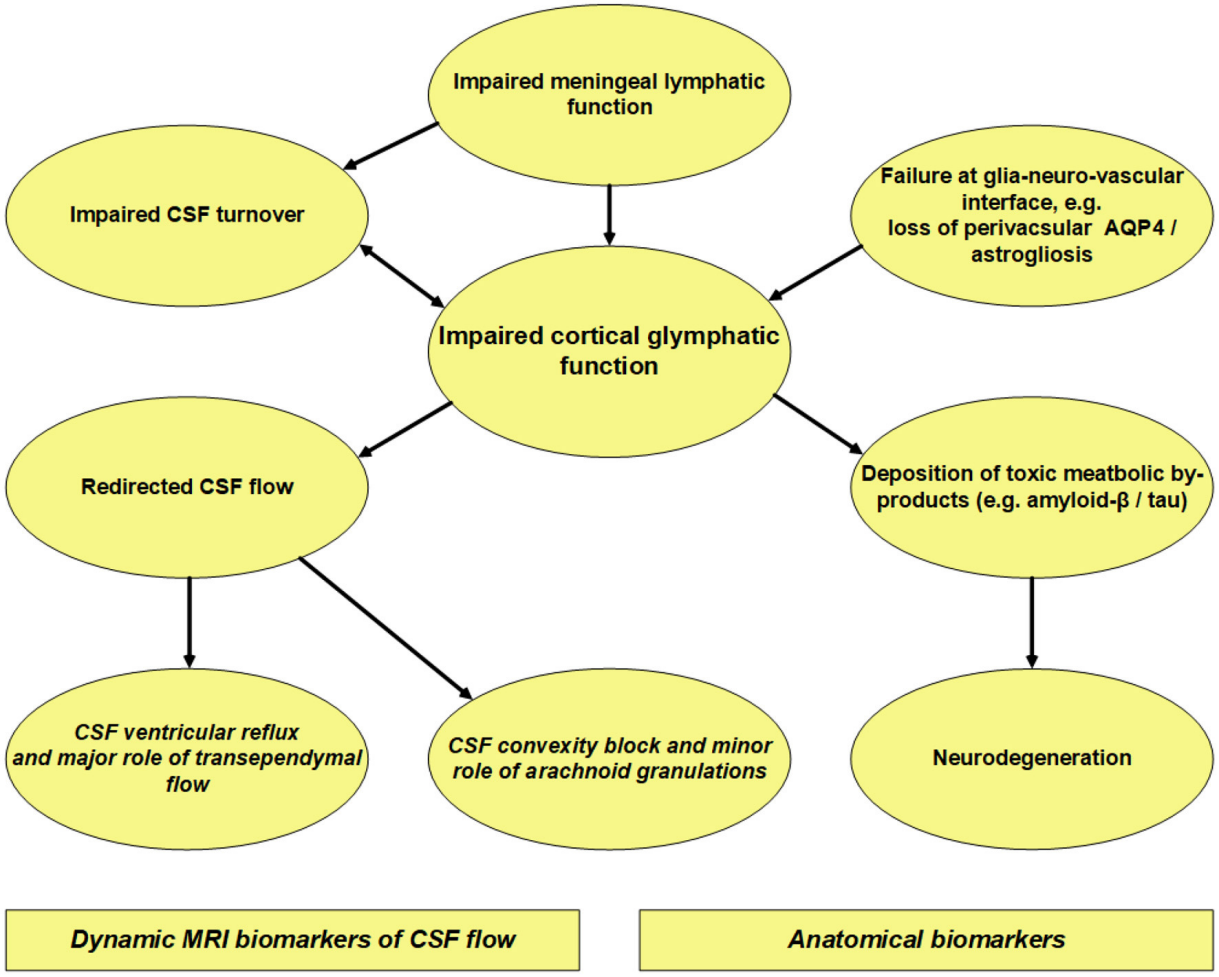
Overview of our current thinking about iNPH pathophysiology based on MRI and histopathological observations in iNPH patients. Concerning intrathecal contrast-enhanced MRI, the contrast agent serves as a tracer within the extra-vascular compartment. This MRI modality showed delayed clearance of tracer from the cerebral cortex (24, 26, 27), which may indicate impaired glymphatic function. Moreover, iNPH demonstrated delayed clearance of tracer from CSF (24, 26) indicative of impaired CSF turnover. Enrichment of tracer in the cerebral cortex was strongly correlated with enrichment in nearby subarachnoid CSF space (24, 26), suggesting that glymphatic clearance heavily depends on molecular clearance from CSF. Histopathological studies in iNPH at cortex show astrogliosis, loss of perivascular AQP4, blood-brain barrier dysfunction, and mitochondrial abnormality (77–81), all of which may affect perivascular solute transport and thereby impair glymphatic function. A typical finding in iNPH is limited and protracted tracer enrichment at cerebral convexity with a peak at 24 h (24), much later than peak concentration in blood (59). This indicates that clearance of tracer from arachnoid granulations to the superior sagittal sinus plays a minor role, though this has traditionally been considered the major efflux route (60). On the other hand, iNPH is characterized by marked tracer reflux to ventricles (grades 3–4) (17, 18), indicative of redistribution of CSF flow to ventricles. Possibly, reversed aqueductal flow with trans-ependymal transport of CSF and further resorption of water through capillary walls may be important in iNPH. We suggest that the meningeal lymphatic vessels serve as the major efflux route for larger molecules within the CSF compartments. Impaired clearance of toxic by-products of brain metabolism due to glymphatic and meningeal lymphatic clearance failure may underlie the deposition of amyloid-β and tau in iNPH (19), and signs of neurodegeneration shown as entorhinal cortex thinning and higher grades of Schelten's MTA and Fazekas scores (18, 26).

The presently described imaging biomarkers provide some information about the pathophysiology behind iNPH, which is further summarized in Figure 10. (1) In iNPH, the flow of CSF is redirected toward the ventricles where transependymal transport of the water and molecular components of CSF may be an essential component. (2) Given the protracted and limited enrichment at a vertex, CSF absorption via arachnoid granulations to the superior sagittal sinus may play a minor role. (3) Delayed clearance from CSF may be instrumental for glymphatic failure. (4) Glymphatic failure affecting the entorhinal cortex may be partaking in the cognitive decline of iNPH patients. (5) In iNPH, defective meningeal lymphatic clearance may be a common cause behind the impaired CSF turnover and glymphatic failure characterizing the disease. Several lines of evidence suggest a crucial role of meningeal lymphatic CSF drainage for removal of cerebral waste products in age-related cognitive decline and Alzheimer's disease (82–84). Experimental data suggest that defective meningeal lymphatic clearance may impair the clearance of neurotoxic metabolites from CSF (85). The meningeal lymphatic drainage capacity becomes impaired with increasing age (83). In humans, the intrathecal MRI contrast agent serving as a CSF tracer passes from subarachnoid CSF to parasagittal dura (38) and to extra-cranial lymph nodes (86).

Some limitations of this study should be noted. At intrathecal enhanced MRI, contrast-induced T1 signal increase is an expression of an increased amount of contrast agent, but necessarily not proportional to changes in concentration. For that, T1 maps would have been necessary, which was beyond the scope of this study. In addition to different magnetic field strengths at 1.5T and 3T, respectively, the T1 gradient echo also differed. Regarding ventricular reflux grading, the imaging routine at 1.5T did not allow for categorizing patients into grades 1 or 2, however, did 9/10 iNPH have grades 3–4 at 1.5T and 3T combined. Furthermore, the study does not incorporate observations from using doses of 0.10 mmol and 0.25 mmol at 3T, and particularly the utility of reducing the dose to 0.10 mmol at 3T may be further explored in later assessments. It should also be noted that we at this stage do not adjust intrathecal dose for specific patient characteristics, such as age, height, or weight. Such adjustments seem meaningful for further dose optimization, and preferably dose reduction. Furthermore, the present data included both individuals with “possible” and “probable” iNPH. Theoretically, there might be differences between these diagnosis sub-categories. However, the one aspect differentiating these categories are the demonstration of normal CSF pressure. It is unlikely that any of our individuals with “possible” iNPH had non-recognized high CSF pressure (i.e., high-pressure hydrocephalus). In addition, the current classification has limitations making distinct differentiation between the possible and probable categories difficult (87).

## Conclusions

Intrathecal gadobutrol can be utilized to trace extra-vascular molecular clearance from the brain and CSF and provide diagnostic information about impaired CSF flow at the brain surface in parallel. A dose of 0.25 mmol maintains adequate diagnostic information about dynamic CSF flow biomarkers (i.e., ventricular reflux grade and glymphatic enhancement) at 1.5T and improves the safety margin compared to 0.50 mmol. A dose of 0.10 mmol was considered insufficient at 1.5T MRI because of too low enrichment in CSF at a vertex and too low glymphatic enhancement in the cerebral cortex and subcortical white matter. Utility of 0.10 mmol at 3T remains to be determined. Strong reflux of tracer to ventricles (grades 3–4) characterizes patients with iNPH, with redirection of CSF flow toward ventricles accompanied with ventricular tracer enrichment. Tracer enrichment at the vertex is slow, with a peak at 48 h, indicating CSF clearance to occur mainly along other pathways than arachnoid granulations. The degree of glymphatic tracer enrichment within the cerebral cortex and subcortical white matter correlates strongly with enrichment within nearby CSF. In particular, we show that this is the case for the entorhinal cortex, which degenerates early in dementia, and where reduced tracer clearance is previously shown to be associated with reduced thickness.

## Data Availability Statement

The raw data supporting the conclusions of this article will be made available by the authors, without undue reservation.

## Ethics Statement

The studies involving human participants were reviewed and approved by the Regional Committee for Medical and Health Research Ethics (REK) of Health Region South-East, Norway (2015/96). The patients/participants provided their written informed consent to participate in this study.

## Author Contributions

PE and GR: conceptualization and design. AL, ØG, BN, and RS: intrathecal injection procedure. ÅH-K, GL, and SV: data management. PE, AP, LV, and GR: data analysis. PE: writing—original draft, supervision and administration, and correspondence and material requests. PE, AL, ÅH-K, ØG, BN, RS, GL, SV, AP, LV, and GR: review and editing and approval of the final manuscript. All authors contributed to the article and approved the submitted version.

## Funding

This work was supported by grants from Health South-East, Norway (Grants 2020068), and from the Department of neurosurgery and The Intervention Centre, Oslo University hospital-Rikshospitalet, Oslo, Norway.

## Conflict of Interest

The authors declare that the research was conducted in the absence of any commercial or financial relationships that could be construed as a potential conflict of interest.

## Publisher's Note

All claims expressed in this article are solely those of the authors and do not necessarily represent those of their affiliated organizations, or those of the publisher, the editors and the reviewers. Any product that may be evaluated in this article, or claim that may be made by its manufacturer, is not guaranteed or endorsed by the publisher.

## Abbreviations

CSF: Cerebrospinal fluid
DESH: Disproportional enlarged subarachnoid space hydrocephalus
GM: Gray matter
iNPH: Idiopathic normal pressure hydrocephalus
ICP: Intracranial pressure
MRI: Magnetic resonance imaging
MTA: Medial temporal atrophy
MMS: Mini-mental state
WM: White matter.
